# From plate to pillow, and vice versa: diet-sleep dynamics in free-living adults with obesity

**DOI:** 10.1007/s00394-026-03894-z

**Published:** 2026-02-16

**Authors:** Juan J. Martin-Olmedo, Antonio Clavero-Jimeno, Jairo H. Migueles, Alba Camacho-Cardenosa, Carmen Piernas, Jonatan R. Ruiz, Lucas Jurado-Fasoli

**Affiliations:** 1 Department of Physiology, Faculty of Medicine, Sport and Health University Research Institute (iMUDS), University of Granada, 18016 Granada, Spain; 2 Department of Physical Education and Sports, Faculty of Sport Sciences, Sport and Health University Research Institute (iMUDS), University of Granada, 18071 Granada, Spain; 3 Instituto de Investigación Biosanitaria de Granada (Ibs.GRANADA), Granada, Spain; 4 Sport and Health University Research Institute (iMUDS), University of Granada, Granada, Spain; 5 Department of Biochemistry and Molecular Biology II, Faculty of Pharmacy, Centre for Biomedical Research, Institute of Nutrition and Food Technology, Universidad de Granada, Granada, Spain; 6 Centro de Investigación Biomédica en Red: Fisiopatología de La Obesidad y Nutrición (CIBEROBN), Instituto de Salud Carlos III, Madrid, Spain

**Keywords:** Sleep pattern, Macronutrients, Food groups, Accelerometry

## Abstract

**Purpose:**

To investigate whether dinner dietary intake is associated with subsequent sleep parameters, and whether sleep parameters are associated with subsequent breakfast dietary intake.

**Methods:**

This study used baseline data from the TEMPUS randomized controlled trial. Eligible participants were adults with obesity (30–40 kg/m^2^; 25–65 years). Sleep parameters were objectively assessed via accelerometry over 14 days. During this time, dietary intake at dinner and breakfast was assessed using one to two 24 h recalls. Dinner dietary intake was matched with sleep registries of the corresponding night, and sleep parameters with the following breakfast dietary intake. Spearman correlation analyses and linear mixed models were used to assess these relationships.

**Results:**

A total of 146 participants (47% women) with valid data were included in the analysis (178 dinner-sleep, and 180 sleep-breakfast observations). Higher carbohydrate, sugars, blue fish, and olive oil intake at dinner were associated with improved subsequent sleep parameters (all *p* ≤ 0.042). In contrast, greater energy, fat, cholesterol, protein, alcohol, red meat, and french fries were associated with poorer subsequent sleep parameters (all *p* ≤ 0.048). Longer sleep duration was associated with enhanced dietary quality at subsequent breakfast (all *p* ≤ 0.034). Moreover, later sleep offset was independently associated with higher energy intake, and greater wake after sleep onset was independently associated with higher carbohydrate intake at subsequent breakfast in multivariate analyses (all *p* ≤ 0.008).

**Conclusion:**

These findings highlight the complex relationship between sleep and diet in free-living settings that may inform future interventions for obesity management.

**Supplementary Information:**

The online version contains supplementary material available at 10.1007/s00394-026-03894-z.

## Introduction

The pandemic of obesity is a major public health concern [1]. Obesity is closely linked to cardiometabolic disorders, such as hypertension, dyslipidemia, and type 2 diabetes mellitus [2, 3]. Increased neck, intra-abdominal, and central adiposity contribute to sleep disorders such as insomnia or obstructive sleep apnea in individuals with obesity [4]. Furthermore, disruptions in sleep patterns are increasingly recognized as risk factors for obesity [4], thereby potentially exacerbating cardiometabolic risk. Thus, elucidating and addressing the bidirectional relationship between obesity and sleep is of paramount public health importance [5].

Sleep is a cornerstone of overall health and well-being [6]. However, at least one-third of adults in Europe and the United States report obtaining less than the recommended seven hours of sleep per night necessary for optimal health maintenance [7, 8]. Insufficient sleep acts as a metabolic stressor, heightening the risk of obesity and cardiometabolic disorders [9, 10]. Evidence from short-term experimental studies in healthy adults indicates that sleep restriction increases energy intake by 250–350 kcal/day with minimal compensatory changes in energy expenditure, leading to increased abdominal visceral adiposity [11–13]. In this context, breakfast, the first meal following the sleep period, may be particularly sensitive to the influence of sleep from the prior night, though it remains largely unclear. Moreover, short sleep duration attenuates body weight and fat loss during calorie restriction [14] and predicts weight regain during interventional efforts to maintain weight loss [15]. Consequently, insufficient sleep may not only increase the risk of obesity but also hinder its effective management by influencing dietary intake, which underscores the need for further research to clarify the relationship between sleep duration and dietary behavior.

Building upon this bidirectional relationship, emerging evidence suggests that diet may also play a role in modulating sleep quality. Higher consumption of fruits, vegetables, whole grains, legumes, olive oil, and fish, and lower intake of processed and sugary foods are associated with improved sleep quality [16]. Additionally, the acute intake of carbohydrates along with tryptophan-rich foods, such as pork, beef, lamb, nuts, seeds, whole grains, and legumes, may enhance sleep quality by promoting the biosynthesis of serotonin and melatonin [17]. Given that dinner directly precedes the nocturnal sleep period, the composition of this meal may exert a particularly relevant influence on subsequent sleep. However, most sleep parameters are derived from self-reported data at the person level rather than on a day-level basis, which limits our ability to ascertain the directionality of the associations between dietary exposure and sleep quality [16]. As a result, the potential role of diet in sleep to enhance obesity management remains unclear.

In contrast to findings from controlled experimental studies, little is known about the relationship between sleep and dietary intake in free-living adults with obesity. In real-world settings, individuals engage in their usual daily routines, and multiple interacting factors may influence both sleep and dietary intake. Consequently, free-living assessments could capture individuals in a broader and more ecologically valid representation of human behavior, thereby enhancing the generalizability of findings. Finally, evaluating dietary intake at dinner and breakfast on a day-level basis, beyond whole-day energy and macronutrient consumption and its relationship with sleep parameters, may provide more nuanced insights into the reciprocal influences of diet and sleep. Thus, this study aimed to determine whether dietary intake at dinner is associated with subsequent sleep parameters, and whether sleep parameters are associated with subsequent breakfast dietary intake in free-living adults with obesity.

## Methods

### Study design

This observational cross-sectional study used data from the baseline assessments of the TEMPUS randomized controlled trial (ClinicalTrials.gov ID: NCT05897073) [18]. The trial was approved by the Granada Provincial Research Ethics Committee (ref. CEI Granada—0365-N-23). Additional details regarding the study design are described elsewhere [18]. During the two-week lead-in period prior to the intervention, participants were instructed to wear an accelerometer for 14 consecutive days (24 h/day), and report dietary intake through non-consecutive 24 h dietary recalls [19]. This study adheres to the STROBE guidelines (Table S1) [20]. The study protocol and experimental design were applied following the last revised ethical guidelines of the Declaration of Helsinki. The study was carried out at the Sport and Health University Research Institute and the San Cecilio and Virgen de las Nieves University Hospital of the University of Granada.

### Participants

Potential participants were recruited in fourteen consecutive waves of 10 to 15 participants from 2nd May 2023 to 27th April 2024. Men and women were recruited from newspaper advertisements, the Endocrinology and Nutrition Unit of the San Cecilio and Virgen de las Nieves University Hospitals of Granada, and the University of Granada community. Inclusion criteria were (i) a body mass index (BMI) ranging from ≥ 30 to < 40 kg/m^2^, (ii) a habitual eating window of at least 11 h, and (iii) stable body weight (within 3% of screening weight) during the preceding 2 months. Exclusion criteria included a diagnosis of diabetes, cardiovascular disease, major sleep or eating disorders, or any significant medical condition that could interfere with or be exacerbated by exercise; shift workers engaged in nocturnal hours; pregnancy or lactation; or participation in a weight loss or a supervised exercise program (1). Before enrollment, potential participants underwent clinical and physical examination assessments to verify their eligibility. Eligible participants received detailed study information and provided informed consent, and those who decided to participate completed the lead-in assessments (Fig. S1).

To better understand how metabolic health status—potentially reflecting differences in metabolic stress—may influence the relationship between dietary intake and sleep, participants were classified as metabolically healthy individuals with obesity (MHO) or metabolically unhealthy participants with obesity (MUO) according to previously established criteria [21]. MUO was defined as having a BMI ≥ 30 kg/m^2^ accompanied by at least one of the following comorbidities: a) fasting triglyceride levels ≥ 150 mg/dL; b) fasting high-density lipoprotein cholesterol (HDL-C) levels < 40 mg/dL for men and < 50 mg/dL for women; c) resting systolic blood pressure ≥ 130 mmHg or diastolic blood pressure ≥ 85 mmHg; d) fasting glucose levels > 100 mg/dL; or e) current medication for triglycerides, cholesterol, blood pressure, or glucose disorders. MHO was defined as having a BMI ≥ 30 kg/m^2^ and none of the above-mentioned risk factors.

To examine how meal timing—an important factor influencing metabolic health and disease risk—may affect the relationship between dietary intake and sleep, participants were categorized based on the timing of their meals. Specifically, participants were classified as early or late dinner eaters based on whether dinner was consumed before or after 21:30 in dinner-sleep observations, and as early or late breakfast eaters if breakfast was consumed before or after 9:00 in sleep-breakfast observations [22]. Lastly, as sleep duration may also modify the relationship between dietary intake and sleep, participants were classified as normal or short sleepers if their total sleep time was ≥ 6 or < 6 h, respectively, in both dinner-sleep and sleep-breakfast observations [23].

### Sleep parameters and dietary intake

During the two-week lead-in period before the intervention, participants were instructed to maintain their habitual dietary, sleep, and physical activity patterns. Participants wore a triaxial accelerometer (ActiGraph GT3X + , ActiGraph LLC, Pensacola, Florida) on their non-dominant wrist to objectively monitor their daily physical activity and sleep patterns throughout the lead-in period. The accelerometers were configured to record raw acceleration data at a frequency of 100 Hz, 24 h per day, for 14 consecutive days. Participants logged their bedtime and wake-up times each day in a tailored mobile phone application for the study (Tempus: com.nnbi.app_extreme_granada; NNBi2020 S.L., Navarra, Spain).

After the two-week lead-in period finished, raw accelerometer data were downloaded using the ActiLife v.6.13.6 software (ActiGraph, Pensacola, FL, USA), and subsequently processed with the open-source R package GGIR [24]. Briefly, the Euclidean Norm of the raw accelerations Minus One (ENMO) with negative values rounded to zero was calculated over 5-s epochs. Non-wear periods were identified based on the magnitude and variability of the raw accelerations measured at each axis [25] and, when appropriate, imputed by the average ENMO at the same time interval during the rest of the recording days. Sleep and wake periods were determined using an automated algorithm based on the variability of the arm posture and guided by the sleep times reported by the participants [25, 26]. This process yielded information on sleep onset, sleep offset, sleep period time (i.e., time from sleep onset to sleep offset), total sleep time (i.e., the amount of time classified as sleep within the sleep period time), total awake time within the sleep period time (wake after sleep onset, WASO), number of awakenings during the sleep period time, and sleep efficiency. It should be noted that time in bed was unavailable; therefore, the sleep efficiency parameter reflected the ratio of total sleep time to sleep period time. Only data from participants with ≥ 70% valid recordings in the sleep period time were included in the analyses.

Dietary intake and timing for dinner and breakfast were assessed using one to two non-consecutive 24 h dietary recalls administered through face-to-face or online interviews by trained research nutritionists. These interviews involved a detailed assessment and description of the food consumed, using photographs of portion sizes to improve subjects' food quantification accuracy [27]. Energy and macronutrient group intake for both meals were calculated using the EvalFINUT® software (https://www.finut.org/evalfinut/), which employs the BEDCA (Base de Datos Española de Composición de Alimentos) database. Macronutrients were also calculated as a percentage of energy intake, fiber as fiber density (g/1000 kcal), and carbohydrate intake as carbohydrate to fiber and carbohydrate to sugar ratios. Individual food items were initially classified into 90 distinct food groups and subsequently regrouped into 31 broader food groups with similar dietary characteristics, aiming to simplify data complexity and facilitate the interpretation of results (Table S2) [28].

Finally, dinner dietary intake was paired with the subsequent sleep parameters, and sleep parameters were matched with the following breakfast dietary intake based on date and time. Dinner to sleep onset time was calculated as the difference between sleep onset and dinner time, while the sleep offset to breakfast time was determined by subtracting sleep offset from breakfast time (Fig. S2).

### Anthropometry, body composition, and cardiometabolic risk markers

Anthropometric and body composition variables (weight, height, BMI, fat mass, fat-free mass, and visceral adipose tissue mass) were assessed under standardized conditions. Cardiometabolic risk markers (fasting glucose, insulin, HbA1c, lipid profile, and blood pressure) were measured with automated analyzers. Full methodological details for these variables are provided in the Supplementary Material.

### Statistical analyses

The sample size for the TEMPUS study was calculated based on its primary outcome (change from baseline to 12 weeks in hepatic fat) [18]. As the present study is a secondary analysis using data from the former study, no specific sample size estimation was performed.

Descriptive data are expressed as mean (SD) when normally distributed or median (first–third quartile) when not, unless otherwise specified. Time variables are expressed as hours:minutes (hh:mm). Data normality was checked using histograms, Q-Q plots, box plots, and the Shapiro–Wilk test. We conducted Spearman correlation analyses to evaluate the relationship between dietary intake outcomes at dinner and subsequent sleep parameters and sleep parameters and following breakfast dietary intake, in the dinner-sleep observations and sleep-breakfast observations, respectively. Due to the multicollinearity observed among dietary intake variables and sleep parameters, we did not correct *P*-values for multiple comparisons using methods such as the Benjamini–Hochberg procedure, as it may overadjust the observed correlations [29]. Correlation analyses were performed separately according to sex (men and women), metabolic health status (MHO and MUO), meal timing (early and late eaters), and sleep duration (normal and short sleepers). Correlation analyses were performed using the corrplot R package (version 0.95).

In order to account for the repeated-measure structure of the data, linear mixed models with participants’ identifiers as random intercepts were used to assess the relationship between individual macronutrients at dinner and subsequent sleep parameters (unadjusted models), and subsequently adjusted for possible confounders, including age, sex, BMI, and moderate-to-vigorous physical activity (adjusted models). Sleep parameters were log10-transformed to meet the normality of the residuals in the models, and estimates were back-transformed for visualization to enhance interpretability. Considering the observed multicollinearity between energy and macronutrient intake at dinner (Fig. S3), we adopted the ‘nutrient density model’ [30], in which energy and macronutrients, expressed as percentages of dinner energy intake, were included as predictors in the models. Similarly, linear mixed models with participants’ identifiers as random intercepts were used to examine the relationship between individual sleep parameters and subsequent breakfast dietary intake (unadjusted models), and then adjusted for age, sex, and BMI (adjusted models). Due to multicollinearity between sleep parameters in sleep-breakfast observations (Fig. S4), we conducted a Principal Component Analysis (PCA) to extract illustrative, lower-dimensional representations of sleep patterns while minimizing redundancy. PCA results guided the selection of sleep offset, sleep period time, and WASO as the primary sleep pattern predictors in linear mixed models. Further details on the sleep parameters selection as predictors of linear mixed models can be found in the Supplementary Material. In both dinner-sleep and sleep-breakfast linear mixed models, variance inflation factor analyses confirmed that the selected predictors effectively minimized collinearity. Moreover, interaction analyses were conducted to determine if sex, metabolic health, meal timing, or sleep duration modified the observed results. When a significant interaction was detected, we performed linear mixed models separately within each subgroup. Linear mixed models were conducted employing the lme4 R package (version 1.1–36).

The level of statistical significance was set at *P* < 0.05. All data curation, statistical analyses, and figures were performed using R version 4.4.3 (https://cran.r-project.org/, The R Project for Statistical Computing, Vienna, Austria).

### Patient and public involvement

Patients and/or the public were not involved in the design, or conduct, or reporting, or dissemination plans of this research.

## Results

A total of 187 adults with obesity (93 women) aged 25–65 years old completed the lead-in measurements (Fig. S1). Forty-one participants were excluded from the analyses as they lacked valid dietary and/or accelerometry data (Fig. S1). From the remaining 146 participants (70 women), 178 dinner-sleep and 180 sleep-breakfast observations were obtained, as some participants provided two separate dinner-sleep and sleep-breakfast observations. The characteristics of the participants included are presented in Table 1, and the dietary data of the observations are shown in Table S3.

**Table 1:** Characteristics of the study participants

	All	Men	Women
*Participant-level characteristics*			
Sample, n (%)	146 (100.0)	76 (52.1)	70 (47.9)
MHO, n (%)	48 (32.9)	25 (34.2)	23 (32.9)
Age (years)	49.0 (42.0–55.0)	48.5 (41.8–53.0)	50.5 (43.3–58.8)
Body weight (kg)	97.6 (89.1–106.4)	103.8 (96.9–111.4)	91.6 (84.0–97.8)
Height (m)	1.68 (0.10)	1.75 (0.07)	1.61 (0.06)
BMI (kg/m^2^)	34.6 (31.6–37.4)	33.9 (31.2–37.1)	35.6 (32.9–38.2)
Fat-free mass (kg)	58.6 (50.5–68.8)	67.8 (62.4–73.5)	50.2 (45.5–53.1)
Fat-free mass (%)	59.1 (54.4–66.3)	66.3 (60.6–68.8)	54.4 (51.2–57.6)
Fat mass (kg)	37.1 (31.9–42.9)	33.9 (29.8–39.7)	41.6 (35.4–44.5)
Fat mass (%)	39.5 (32.0–43.8)	32.1 (29.5–37.8)	43.8 (40.6–47.3)
VAT mass (g)	764.4 (655.1–956.6)	800.7 (673.6–970.2)	747.4 (612.2–936.6)
Fasting glucose (mg/dL)	88 (83–95)	89 (84–94)	86 (81–96)
HbA1c (%)	5.5 (0.4)	5.4 (0.3)	5.6 (0.4)
HOMA-IR	2.03 (1.45–2.88)	2.23 (1.69–3.29)	1.92 (1.29–2.38)
Total cholesterol (mg/dL)	203 (36)	202 (38)	205 (35)
HDL-C (mg/dL)	51 (45–58)	48 (42–54)	54 (48–66)
LDL-C (mg/dL)	129 (32)	129 (34)	129 (29)
Triglycerides (mg/dL)	100 (69–132)	111 (76–162)	89 (62–111)
Systolic blood pressure (mmHg)	118 (13)	122 (11)	113 (14)
Diastolic blood pressure (mmHg)	84 (9)	85 (9)	83 (8)
*Sleep parameters from dinner-sleep observations (n* = 178, 49% in women)*			
Early dinner eaters, n (%)^a^	100 (56.2)	51 (56.0)	49 (56.3)
Normal sleepers, n (%)^b^	115 (64.6)	56 (61.5)	59 (67.8)
Dinner to sleep onset time (hours)	2.4 (1.6–3.2)	2.2 (1.6–3.0)	2.5 (1.6–3.5)
Sleep onset (hh:mm)	00:07 (01:17)	00:01 (01:15)	00:12 (01:19)
Sleep period time (hours)	7.5 (6.8–8.4)	7.5 (7.0–8.4)	7.4 (6.6–8.3)
Total sleep time (hours)	6.5 (1.2)	6.5 (1.2)	6.5 (1.3)
Wake after sleep onset (hours)	0.9 (0.6–1.4)	1.0 (0.6–1.5)	0.8 (0.6–1.3)
Number of awakenings (n)	2.0 (0.0–3.0)	2.0 (0.0–4.0)	1.0 (0.0–3.0)
Sleep efficiency (%)	87.6 (81.9–92.1)	86.5 (80.5–91.0)	88.4 (84.1–92.4)
Sleep offset (hh:mm)	07:40 (01:23)	07:38 (01:23)	07:43 (01:23)
MVPA (min/day)	11.0 (0.0–37.3)	10.8 (0.0–32.5)	13.0 (0.0–45.3)
*Sleep parameters from sleep-breakfast observations (n* = 180, 48% in women)*			
Early breakfast eaters, n (%)^a^	83 (46.1)	49 (52.7)	34 (39.1)
Normal sleepers, n (%)^b^	118 (65.6)	58 (62.4)	60 (69.0)
Sleep onset (hh:mm)	00:20 (01:16)	00:23 (01:17)	00:16 (01:13)
Sleep period time (hours)	7.4 (6.7–8.3)	7.3 (6.5–8.3)	7.6 (6.8–8.4)
Total sleep time (hours)	6.4 (1.3)	6.3 (1.3)	6.6 (1.2)
Wake after sleep onset (hours)	0.9 (0.5–1.4)	1.0 (0.5–1.4)	0.9 (0.5–1.3)
Number of awakenings (n)	1.0 (0.0–3.0)	2.0 (0.0–4.0)	1.0 (0.0–3.0)
Sleep efficiency (%)	87.5 (81.8–92.5)	85.4 (81.7–92.5)	88.3 (82.7–92.5)
Sleep offset (hh:mm)	07:47 (01:24)	07:41 (01:17)	07:55 (01:30)
Sleep offset to breakfast time (hours)	1.0 (0.3–2.3)	1.0 (0.3–2.3)	1.0 (0.3–2.3)

Time variables (hh:mm) are expressed as mean (SD). The other variables are presented as mean (SD) when normally distributed or median (first quartile–third quartile) when not, unless otherwise stated

*BMI* body mass index, *HbA1c* glycated haemoglobin, *HDL-C* high-density lipoprotein cholesterol, *HOMA-IR* Homeostatic Model Assessment for Insulin Resistance, *LDL-C* low-density lipoprotein cholesterol, *MHO* metabolically healthy participants with obesity, *VAT* visceral adipose tissue

^*^Some participants contributed with two dinner-sleep and sleep-breakfast observations

^a^Early dinner eaters in dinner-sleep observations were participants whose dinner took place before 21:30, whereas early breakfast eaters in sleep-breakfast observations were participants whose breakfast took place before 9:00

^b^Normal sleepers in dinner-sleep and sleep-breakfast observations were participants whose total sleep time was ≥ 6 h

### Dietary intake at dinner is associated with subsequent sleep parameters

**Fig. 1:**
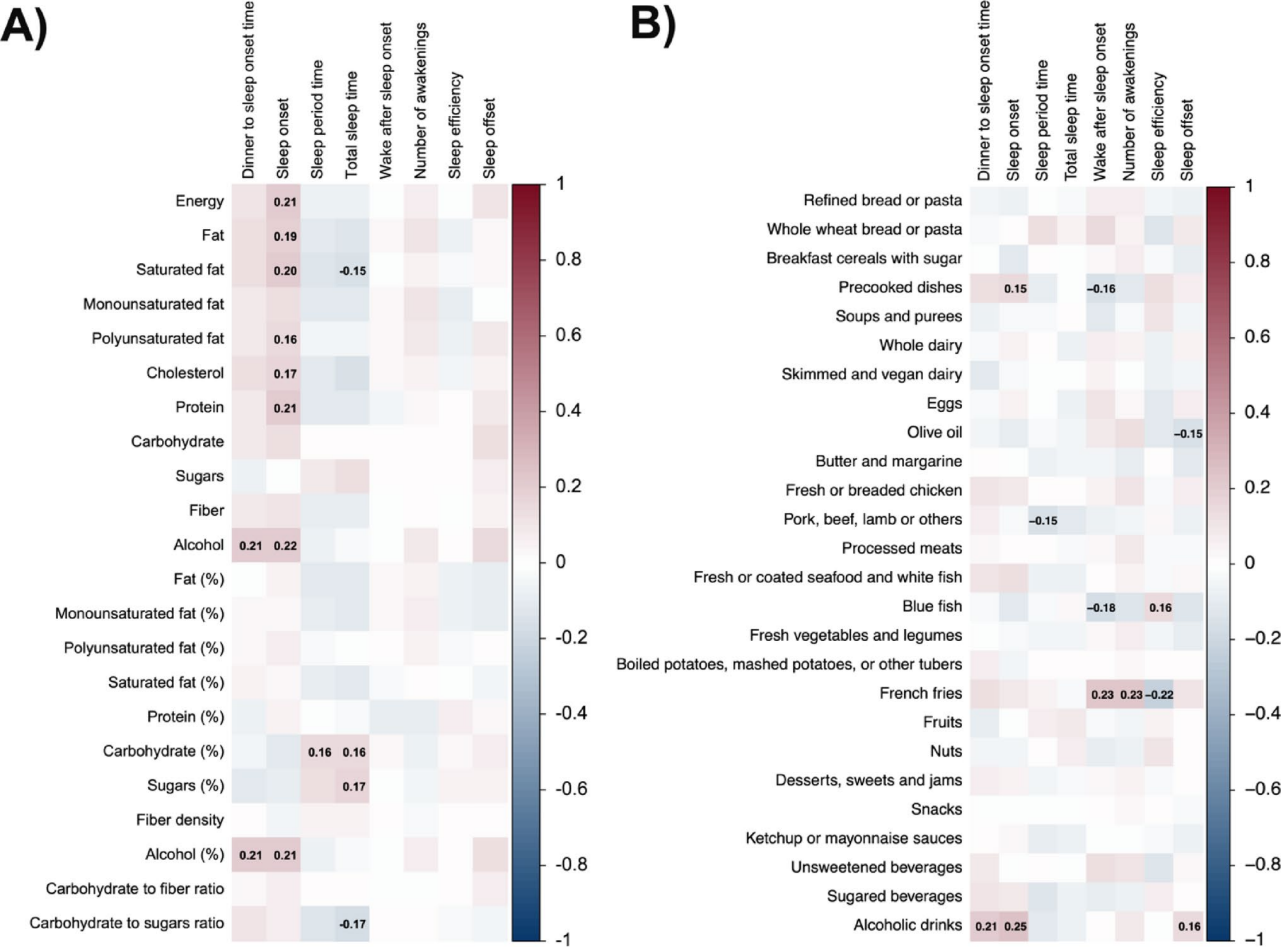
Bivariate correlations between nutrients (panel A) and food groups intake (Panel B) at dinner with subsequent sleep parameters in all participants. The colours of the squares represent the Spearman correlation coefficient. Red colours represent positive Spearman coefficients, whereas blue depicts negative coefficients. Bold numbers inside the squares represent statistically significant Spearman correlation coefficients (*P* < 0.05)

Dinner energy, fat (g), saturated fat (g), polyunsaturated fat (g), cholesterol, protein (g), and alcohol intake (g and %energy) were positively correlated with sleep onset (all ρ ≥ 0.22, *P* ≤ 0.036; Fig. 1A). Moreover, carbohydrate (%energy) and sugars intake (%energy) showed positive associations with total sleep time (all ρ ≥ 0.16, *P* ≤ 0.034), whereas saturated fat (g) and total carbohydrate to total sugars ratio were inversely associated with total sleep time (both ρ ≤ − 0.15, *P* ≤ 0.047) (Fig. 1A). Dinner blue fish consumption was positively correlated with sleep efficiency (ρ = 0.16, *P* = 0.034) and inversely with WASO (ρ = − 0.18, *P* = 0.019), while french fries consumption was inversely correlated with sleep efficiency (ρ = − 0.22, *P* = 0.003) (Fig. 1B). Dinner olive oil intake was inversely associated with sleep offset (ρ = − 0.15, *P* = 0.042), whereas pork, beef, lamb, or others consumption was inversely associated with sleep period time (ρ = − 0.15, *P* = 0.048; Fig. 1B). Finally, alcoholic drinks were positively associated with dinner to sleep onset time, sleep onset, and sleep offset (all ρ ≥ 0.16, *P* ≤ 0.039; Fig. 1B). Similar patterns of association were observed in subgroup analyses by sex (Fig. S5), metabolic health (Fig. S6), meal timing (Fig. S7), and sleep duration (Fig. S8).

**Fig. 2:**
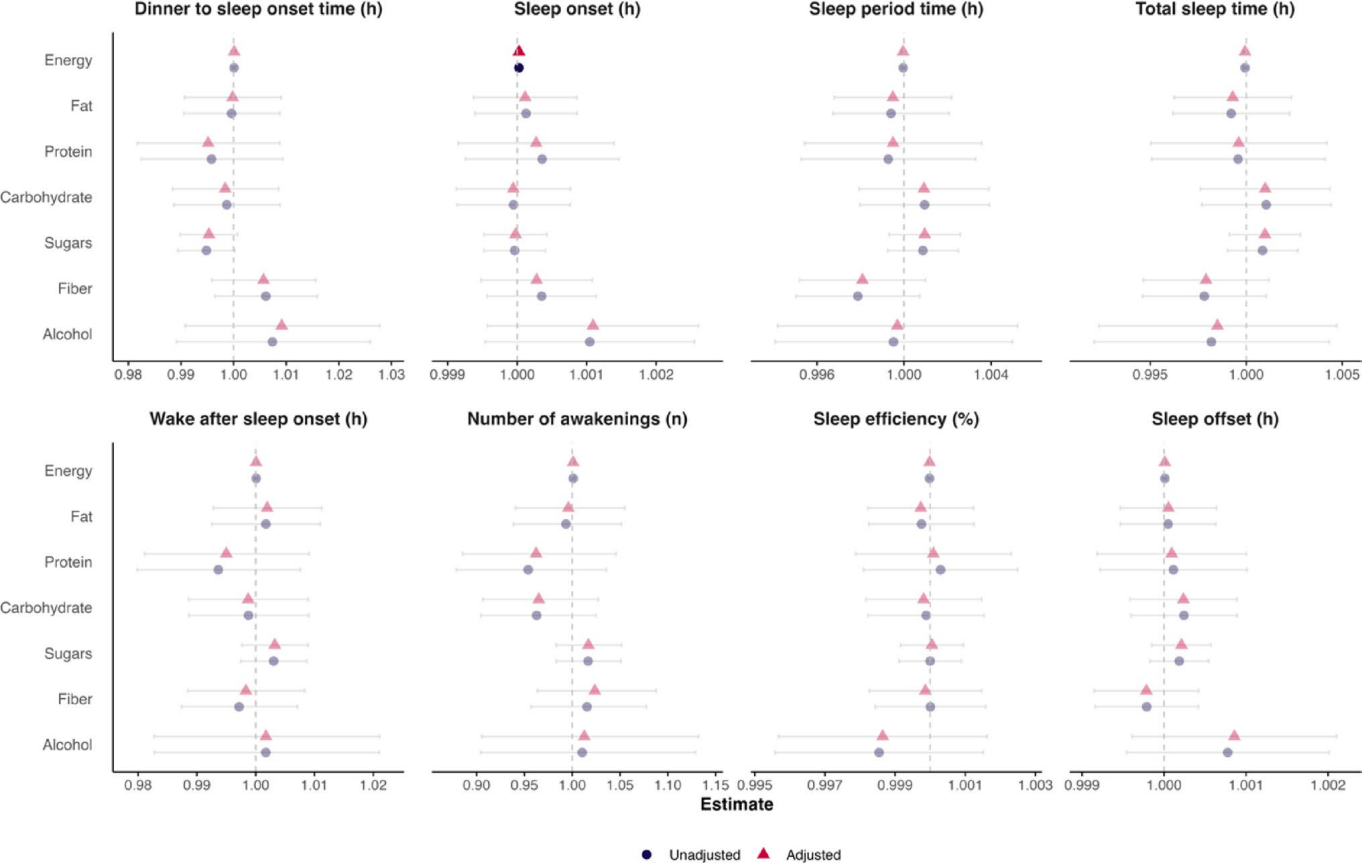
Forest plots of dinner energy intake and macronutrient intake associations with subsequent sleep parameters in all participants. Dietary data were imputed using the nutrient density model, as described elsewhere (1). Estimates and 95% confidence intervals (CIs) were obtained via linear mixed models. Blue circles and their corresponding 95% CIs represent estimates from unadjusted models, whereas red triangles and their corresponding 95% CIs represent estimates from models adjusted for age, sex, body mass index, and moderate-to-vigorous physical activity. Sleep parameters were log10-transformed for statistical analyses; however, results are back-transformed to improve interpretability. Vivid symbols and 95% CIs indicate significant estimates (*P* < 0.05)

Next, we conducted linear mixed models to examine the association between individual macronutrient intake (%energy) at dinner and subsequent sleep parameters, when adjusted for each other and potential confounders. We found no significant associations between individual dinner macronutrients ingested at dinner and subsequent sleep parameters in multivariate analyses in all participants (all *P* ≥ 0.063; Fig. 2), early eaters (all *P* ≥ 0.064; Fig. S10A), and short sleepers (all *P* ≥ 0.062; Fig. S11B). However, higher fat intake at dinner was independently associated with increased WASO in normal sleepers (all *P* ≤ 0.049; Fig. S11A). Greater sugars intake at dinner was independently associated with shorter dinner to sleep onset time in MHO (all *P* ≤ 0.043; Fig. S9A); later sleep offset in MUO (all *P* ≤ 0.028; Fig. S9B); and higher WASO, number of awakenings, and lower sleep efficiency in late eaters (all *P* ≤ 0.039; Fig. S10B). Higher fiber consumption was independently associated with longer dinner to sleep onset time in MHO (*P* = 0.031; Fig. S9A), but this significant association was lost after adjustment for confounders (*P* = 0.050; Fig. S9A). Higher fiber intake was independently associated with greater dinner to sleep onset time and lower sleep period and total sleep time, but also lower WASO in late eaters (all *P* ≤ 0.040; Fig. S10B), and lower total sleep time in normal sleepers (*P* = 0.039; Fig. S11A). No interaction by sex was detected in the multivariate analyses.

### Sleep parameters may influence dietary intake at subsequent breakfast

**Fig. 3:**
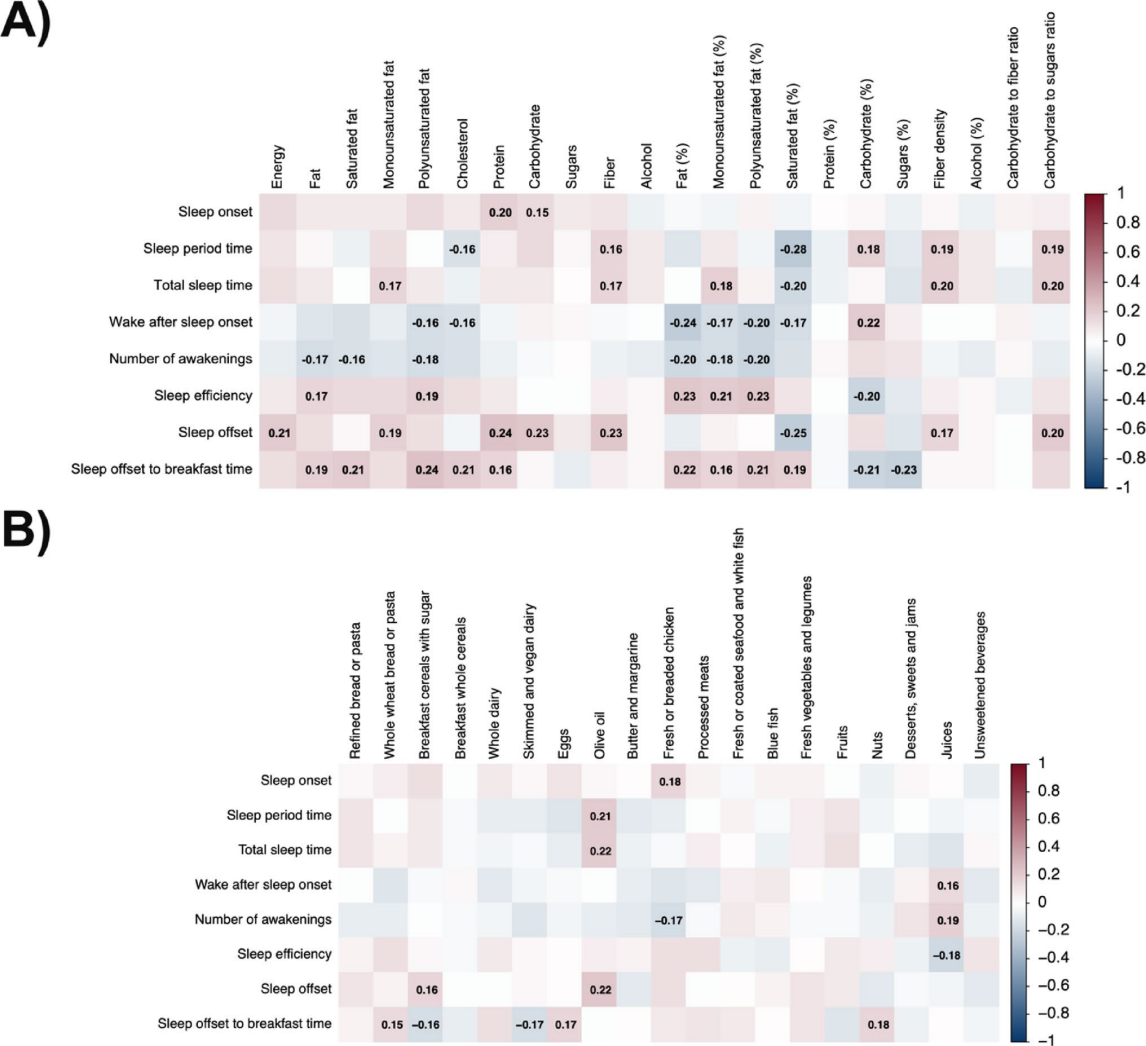
Bivariate correlations between sleep parameters and nutrients (panel A) and food groups intake (Panel B) at subsequent breakfast in all participants. The colours of the squares represent the Spearman correlation coefficient. Red colours represent positive Spearman coefficients, whereas blue depicts negative coefficients. Bold numbers inside the squares represent statistically significant Spearman correlation coefficients (*P* < 0.05)

Sleep duration (i.e., sleep period and/or total sleep time) was positively correlated with breakfast monounsaturated fat (g and %energy), fiber (g), fiber density (g/1000 kcal), carbohydrate (%energy), carbohydrate to sugar ratio, and olive oil intake (all ρ ≥ 0.16, *P* ≤ 0.030; Fig. 3A, B). Conversely, sleep duration was inversely associated with cholesterol and saturated fat intake (%energy) (all ρ ≤ -0.16, *P* ≤ 0.034; Fig. 3A). Sleep continuity (i.e., greater sleep efficiency, and/or lower WASO and number of awakenings) was positively associated with breakfast fat (g and %energy), saturated fat (g and %energy), monounsaturated fat (%energy), and polyunsaturated fat intake (g and %energy), and inversely associated with carbohydrate (%energy) and juices consumption (all *P* ≤ 0.033; Fig. 3A, B). Sleep onset was positively associated with protein (g), carbohydrate (g) and fresh or breaded chicken consumption (all ρ ≥ 0.15, *P* ≤ 0.039; Fig. 3A, B). Sleep offset was positively associated with breakfast energy, monounsaturated fat (g), protein (%energy), carbohydrate (g), fiber (g), fiber density (g/1000 kcal), carbohydrate to sugar ratio, breakfast cereals with sugar and olive oil consumption (all ρ ≥ 0.16, *P* ≤ 0.029; Fig. 3A, B), and inversely associated with saturated fat (%energy) intake (ρ = − 0.25, *P* < 0.001; Fig. 3A). Similar patterns of association were observed in subgroup analyses by sex (Fig. S12), metabolic health (Fig. S13), meal timing (Fig. S14), and sleep duration (Fig. S15).

**Fig. 4:**
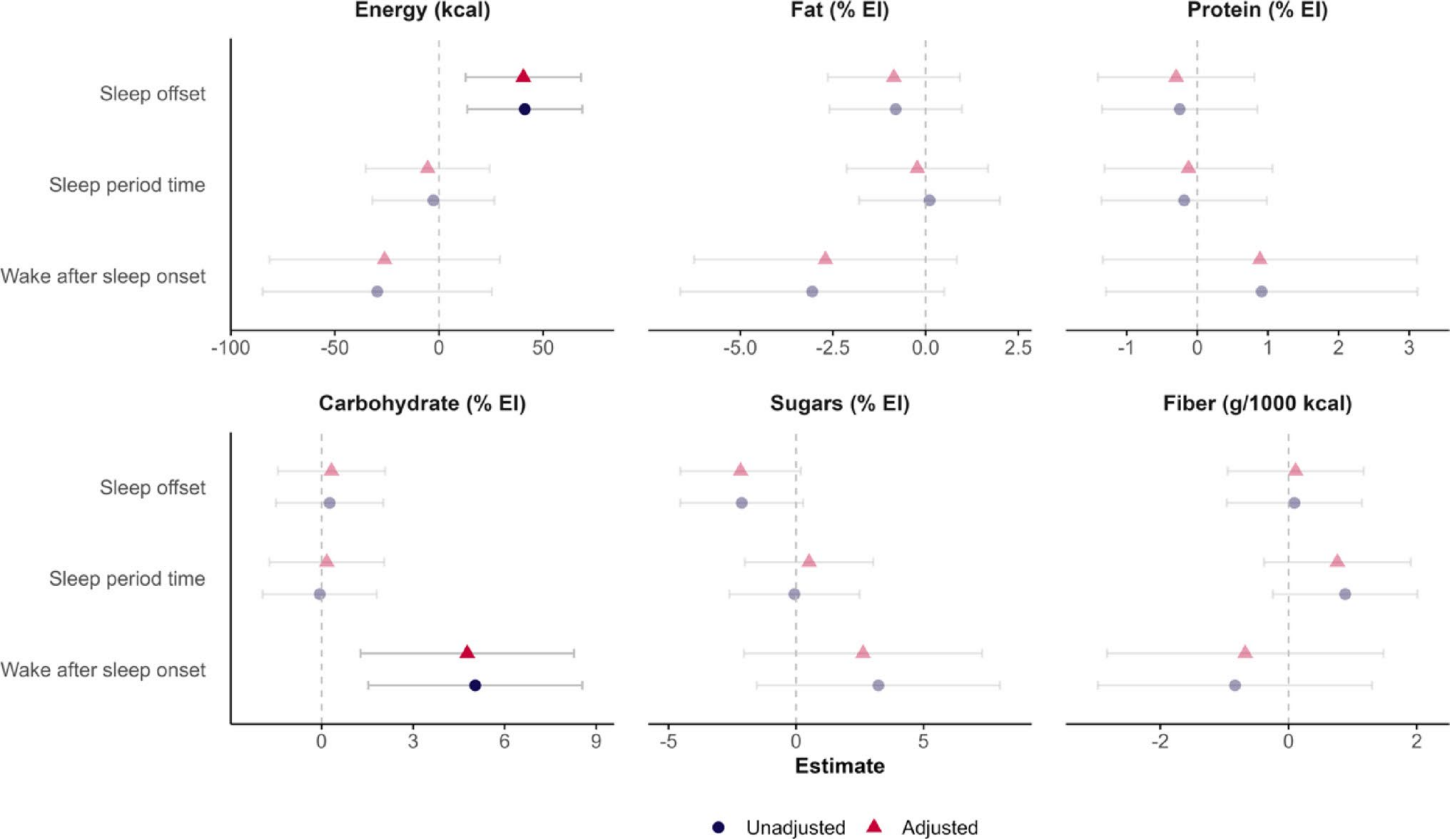
Forest plots of associations of sleep parameters with subsequent breakfast energy and macronutrient intake in all participants. Dietary data were imputed using the nutrient density model, as described elsewhere (1). Estimates and 95% confidence intervals (CIs) were obtained via linear mixed models. Blue circles and their corresponding 95% CIs represent estimates from unadjusted models, whereas red triangles and their corresponding 95% CIs represent estimates from models adjusted for age, sex, and body mass index. Vivid symbols and 95% CIs indicate significant estimates (*P* < 0.05)

We observed that later sleep offset was independently associated with higher energy intake, and greater WASO was independently associated with higher carbohydrate intake at subsequent breakfast in multivariate analyses in all participants (all *P* ≤ 0.008; Fig. 4). Similar associations were found in women (all *P* ≤ 0.035; Fig. S16B) but not in men (all *P* ≥ 0.060; Fig. S16A). No interaction of metabolic health, meal timing, or sleep duration was detected in the multivariate analyses. These results were consistent after adjusting for overnight fasting (data not shown).

## Discussion

This study comprehensively shows the association of dietary intake at dinner with subsequent sleep parameters and the association of sleep on subsequent dietary intake at breakfast in free-living adults with obesity. Our results suggest that dietary intake at dinner is weakly associated with subsequent sleep quality, and that sleep quality, in turn, may influence energy and macronutrient intake at subsequent breakfast. These findings highlight the complex and dynamic interplay between sleep and dietary intake in free-living settings that holds potential to inform future interventions targeting obesity management.

Our findings show that greater intake of energy, fat, cholesterol, protein, alcohol, red meat, and french fries are associated with poorer subsequent sleep quality. Pro-inflammatory diets—characterized by high energy, saturated fat, cholesterol, protein, red meat, or processed foods consumption, such as french fries—have been associated with impaired sleep parameters [31]. A pro-inflammatory dietary intake at dinner may interfere with the circadian secretion of inflammatory cytokines and hormones, including cortisol and melatonin, thereby compromising subsequent sleep [32]. Conversely, consuming polyunsaturated fat, olive oil, and blue fish at dinner may exert acute anti-inflammatory effects, enhancing subsequent sleep parameters by supporting dopaminergic and serotoninergic neurotransmission [16, 33]. Moreover, carbohydrate consumption at dinner, particularly from high-glycemic index foods, may acutely improve sleep duration and sleep onset latency by augmenting tryptophan brain availability, which may facilitate serotonin and melatonin biosynthesis [17, 34, 35]. Alcohol intake at dinner may impair sleep, an adverse effect likely mediated by the accumulation of alcohol secondary metabolites, such as acetaldehyde and acetate, which can increase physiological arousal and body temperature [36], reducing the rapid-eye-movement (REM) sleep phase [37], as well as potentially prolonging dinner to sleep onset intervals of time and delaying sleep onset, as observed in the present study. Taken together, these findings suggest that both dinner macronutrient composition and specific food groups are associated with subsequent sleep quality in adults with obesity.

We observed no significant associations between dinner macronutrient intake and subsequent sleep parameters in multivariate analyses, suggesting that macronutrient intake at dinner does not substantially influence sleep quality in this population. These findings align with prior observational and intervention studies, which yielded inconclusive results regarding the impact of whole-day dietary macronutrient intake on sleep [34, 35]. However, in specific subgroup analyses, we revealed that higher fat intake at dinner was associated with greater WASO in normal sleepers. Similar results have been reported in intervention studies, where higher whole-day fat consumption, particularly saturated fat, increases WASO and shortens REM sleep phase in normal sleepers [38, 39]. These effects may be mediated by postprandial elevations in cholecystokinin, which may modulate sleep architecture [38]. Furthermore, greater sugars intake (%energy) at dinner was independently associated with shorter dinner to sleep onset time in MHO, but with later sleep offset in MUO and reduced sleep continuity in late eaters. It is plausible that impaired nocturnal glucose metabolism in both MUO and late eaters underlies these divergent associations as compared with MHO [22, 40]. Additionally, we observed that higher fiber intake was generally associated with poorer sleep parameters in late eaters and normal sleepers. However, acute responses to fiber intake at dinner may not necessarily reflect long-term effects of its consumption. For instance, whole-day high-fiber diets have been associated with greater sleep continuity, increased REM sleep phase, and shorter sleep latency in different populations [41]. Long-term fiber ingestion may exert anti-inflammatory effects by promoting short-chain fatty acids production that potentially enhances sleep parameters [16]. Overall, these findings underscore the complexity of the relationship between dinner macronutrient composition and sleep and suggest that this association may vary according to metabolic health status, dinner timing, and sleep duration. Further research should confirm these results using different criteria for defining early and late eaters, and normal or short sleepers.

Overall, longer sleep duration was associated with improved dietary quality at subsequent breakfast. These findings are in line with previous observational and intervention studies reporting that shorter sleep duration is associated with poorer whole-day diet quality (e.g., lower intake of fruits and vegetables, and higher absolute and relative fat intake) in both healthy individuals and adults with obesity [42, 43]. Interestingly, although we did not detect significant associations between sleep duration and energy intake in free-living adults with obesity, some randomized controlled trials have found that sleep restriction increases whole-day energy intake in both healthy adults and adults with obesity [11–13], while sleep extension reduces whole-day energy intake in adults with overweight [44]. It could be that potential mechanisms promoting overeating after sleep restriction, such as increased hunger, alterations in appetite-regulating hormones as leptin or ghrelin, and intensified reward-seeking behavior [40], may be attenuated in free-living settings compared to tightly controlled conditions, or that whole-day effects may be greater compared to solely the breakfast intake.

In our study, sleep continuity and timing were also associated with subsequent dietary intake at breakfast, yet yielded complex, mixed results that do not delineate whether enhancing these sleep dimensions improves subsequent breakfast dietary quality. Despite these varied associations, we found that each additional hour of later sleep offset was independently associated with a ~ 40 kcal increase in subsequent breakfast energy intake, irrespective of sleep period time and WASO. Similarly, each additional hour of WASO corresponded to a ~ 5% energy intake elevation from carbohydrate intake in the subsequent breakfast, independent of sleep offset and sleep period time. Increased WASO was also independently associated with lower fiber and higher sugar intake at breakfast, although this association was not statistically significant. Overall, these results suggest that the higher breakfast carbohydrate intake observed with greater WASO may be characterized by a relatively greater contribution from sugar and a lower contribution from fiber. Later sleep offset may induce acute circadian disruptions that potentially impact levels of hunger-regulating hormones and reward-seeking behavior, likely increasing ad libitum energy intake in adults with obesity [45, 46]. However, it is currently unknown if these mechanisms may mediate the association between sleep continuity and dietary intake. Notably, similar associations were detected in women, but not in men, indicating a potential sex dimorphism in the effects of sleep patterns on subsequent dietary intake at breakfast. Overall, the herein reported associations may provide valuable insights to be confirmed by future studies assessing the impact of objectively assessed sleep timing or continuity on subsequent dietary intake in this population.

The present study has several strengths and limitations. A key strength is the focus on meal-specific associations (i.e., dinner-sleep and sleep-breakfast observations) in a day-level basis, which may help clarify the directionality and temporal dynamics of these associations and inform the development of targeted dietary and lifestyle interventions in future research. Moreover, the objective evaluation of sleep parameters in free-living adults with obesity ensures that our findings more accurately reflect real-life conditions, thereby enhancing their potential generalizability within this population. However, the cross-sectional design precludes causal inferences. Another limitation is the inclusion of only adults with obesity, which restricts the extrapolation of the findings to other populations such as children, older adults, shift workers, or those with additional health conditions. Additionally, this study presents exploratory findings, and several of the observed associations were of relatively small magnitude, which should be considered when evaluating their clinical relevance. Lastly, dietary intake was self-reported, and we were unable to derive reliable dietary patterns from dinner and breakfast meals to examine the relationship between representative combinations of food groups and sleep parameters.

## Conclusion

This study shows that dinner dietary intake is weakly associated with subsequent sleep quality, while sleep quality is associated with subsequent breakfast dietary intake in free-living adults with obesity. Moreover, this reciprocal relationship could vary according to metabolic health status, meal timing, and sleep duration within this population. These findings underscore the complex relationship between sleep and diet in free-living settings and inform future interventions for obesity management.

## Supplementary Information

Below is the link to the electronic supplementary material.

Supplementary Material 1

## Data Availability

The data that support the findings of this study are available from the corresponding authors, JJM-O and JRR, upon reasonable request.
