# Supplementary material for: From plate to pillow, and vice versa: diet-sleep dynamics in free-living adults with obesity
Source: Eur J Nutr. 2026 Feb 16;65(2):63. doi: 10.1007/s00394-026-03894-z (PMC12909376; doi:10.1007/s00394-026-03894-z)
Supplement: Supplementary file 1 — Supplementary Material 1 [file 394_2026_3894_MOESM1_ESM.docx]

**From Plate to Pillow, and Vice Versa: Diet-Sleep Dynamics in Free-Living Adults with Obesity**

**Supplementary Material,** including:

- Supplementary methods: Anthropometrics and body composition.
- Supplementary methods: Cardiometabolic risk markers.
- Supplementary methods: Sleep parameters selection as predictors for linear mixed models.
- Table S1. STROBE checklist.
- Table S2. Main foods included in each food group category.
- Table S3. Dietary data of the study participants.
- Figure S1. Study flow diagram.
- Figure S2. Overview of the study design.
- Figure S3. Bivariate correlations between energy and macronutrient intake at dinner.
- Figure S4. Bivariate correlations between sleep parameters before breakfast.
- Figure S5. Bivariate correlations between nutrients and food groups intake at dinner with subsequent sleep parameters in men and women.
- Figure S6. Bivariate correlations between nutrients and food groups intake at dinner with subsequent sleep parameters in metabolically healthy participants with obesity and metabolically unhealthy participants with obesity.
- Figure S7. Bivariate correlations between nutrients and food groups intake at dinner with subsequent sleep parameters in early dinner eaters and late dinner eaters.
- Figure S8. Bivariate correlations between nutrients and food groups intake at dinner with subsequent sleep parameters in normal sleepers and short sleepers.
- Figure S9. Forest plots of associations of dinner energy intake and macronutrient intake with subsequent sleep parameters in metabolically healthy participants with obesity, and metabolically unhealthy participants with obesity.
- Figure S10. Forest plots of associations of dinner energy intake and macronutrient intake with subsequent sleep parameters in early dinner eaters, and late dinner eaters.
- Figure S11. Forest plots of associations of dinner energy intake and macronutrient intake with subsequent sleep parameters in normal sleepers, and short sleepers.
- Figure S12. Bivariate correlations between sleep parameters and nutrients and food groups intake at subsequent breakfast in men and women.
- Figure S13. Bivariate correlations between sleep parameters and nutrients and food groups intake at subsequent breakfast in metabolically healthy participants with obesity and metabolically unhealthy participants with obesity.
- Figure S14. Bivariate correlations between sleep parameters and nutrients and food groups intake at subsequent breakfast in early breakfast eaters (breakfast earlier than 9:00; panels A and B, respectively) and late breakfast eaters.
- Figure S15. Bivariate correlations between sleep parameters and nutrients and food groups intake at subsequent breakfast in normal sleepers (total sleep time ≥ 6 h; panels A and B, respectively) and short sleepers.
- Figure S16. Forest plots of associations of sleep parameters with subsequent breakfast energy intake and macronutrient intake in men (panel A) and women.

**Supplementary Methods**

*Anthropometrics and body composition*

Weight and height were measured barefoot and wearing light clothing, using a SECA scale and stadiometer (model 799; Electronic Column Scale, Hamburg, Germany), and were used to calculate BMI (kg/m^2^). Body composition was assessed in the morning after an overnight fast by dual-energy X-ray absorptiometry with a Hologic Discovery Wi device (Hologic Inc., Bedford, MA, USA) and analyzed using APEX software (version 4.0.2). The device was calibrated daily using a lumbar spine phantom. Participants were scanned in a supine position, remaining still throughout the procedure. The mean total fat-free mass (kg and percentage), total fat mass (kg and %), and visceral adipose tissue (VAT) mass were obtained from two consecutive whole-body DXA scans.

*Cardiometabolic risk markers*

﻿Venous blood samples were stored at −80°C to preserve their integrity for subsequent analysis. We measured ﻿fasting blood glucose (Alinity C system analyzer, Abbott Laboratories, Illinois, USA), insulin (UniCel DxI 800 access immunoassay system, Beckman Coulter, California, USA), haemoglobin A1c (automated glycohaemoglobin G11 analyser, Horiba) and lipid profile [i.e., total cholesterol, HDL-C, and triglycerides] using Alinity C system analyzer (Abbott Laboratories). Low-density lipoprotein cholesterol (LDL-C) was calculated using a validated equation (LDL-C = total cholesterol – HDL-C – (triglycerides/5)) [1]. ﻿Additionally, systolic and diastolic blood pressure were assessed in the morning using an automated monitor (M3-Comfort, Omron Healthcare Europe B.V., Hoofddorp, The Netherlands) following the guidelines of the 2021 European Society of Hypertension [2].

*Sleep parameters selection as predictors for linear mixed models*

After processing the accelerometry data as described in the Methods section, we derived sleep timing (sleep onset, sleep offset, and sleep offset to breakfast time), sleep duration (sleep period time and total sleep time), and sleep continuity [wake after sleep onset (WASO), number of awakenings, and sleep efficiency] parameters. Sleep timing, duration, and continuity variables in sleep-breakfast observations were strongly correlated (**Fig. S4**). Accordingly, variance inflation factors (VIF) from linear mixed models including all sleep parameters and covariates indicated substantial multicollinearity (VIF values > 10) for all sleep parameters, except number of awakenings and sleep offset to breakfast time (**Table 1**).

| **Table 1.** Variance inflation factor for predictors in the linear mixed models with all predictors. | |
| --- | --- |
| **Predictor** | **VIF** |
| Sleep onset | 301.32 |
| Sleep period time | 2.58E+10 |
| Total sleep time | 2.04E+10 |
| Wake after sleep onset | 5.53E+09 |
| Number of awakenings | 2.68 |
| Sleep efficiency | 28.77 |
| Sleep offset | 364.58 |
| Sleep offset to breakfast time | 1.39 |
| Sex | 1.07 |
| Age | 1.11 |
| BMI | 1.07 |
| Abbreviations: BMI, body mass index; VIF, variance inflation factor. | |

Given this multicollinearity in the sleep-breakfast observations, we conducted a Principal Component Analysis (PCA) to obtain illustrative, lower-dimensional representations of sleep patterns while minimizing redundancy. Our aim was to retain one representative sleep parameter of sleep timing (sleep onset, sleep offset, or sleep offset to breakfast time), sleep duration (sleep period time or total sleep time), and sleep continuity (WASO, number of awakenings, or sleep efficiency). PCA components 1 (PC1), 2 (PC2), and 3 (PC3) explained ~39.9%, 28.6%, and 19.8% of the variance in sleep, respectively (**Figure 1**). Therefore, we selected predictors based on these PCA components and the loadings of each original sleep parameter in these components (**Table 2**). For sleep timing, we retained sleep offset because it was the strongest contributor on both PC1 and PC2. For sleep duration, we retained the sleep period time, which contributed more strongly to PC1 than total sleep time. For sleep continuity, we retained WASO, as it contributed more strongly to PC1 than sleep efficiency and number of awakenings. Therefore, the VIF values calculated over the final models including these three sleep variables and covariates confirm no risk of multicollinearity (**Table 3**).

**
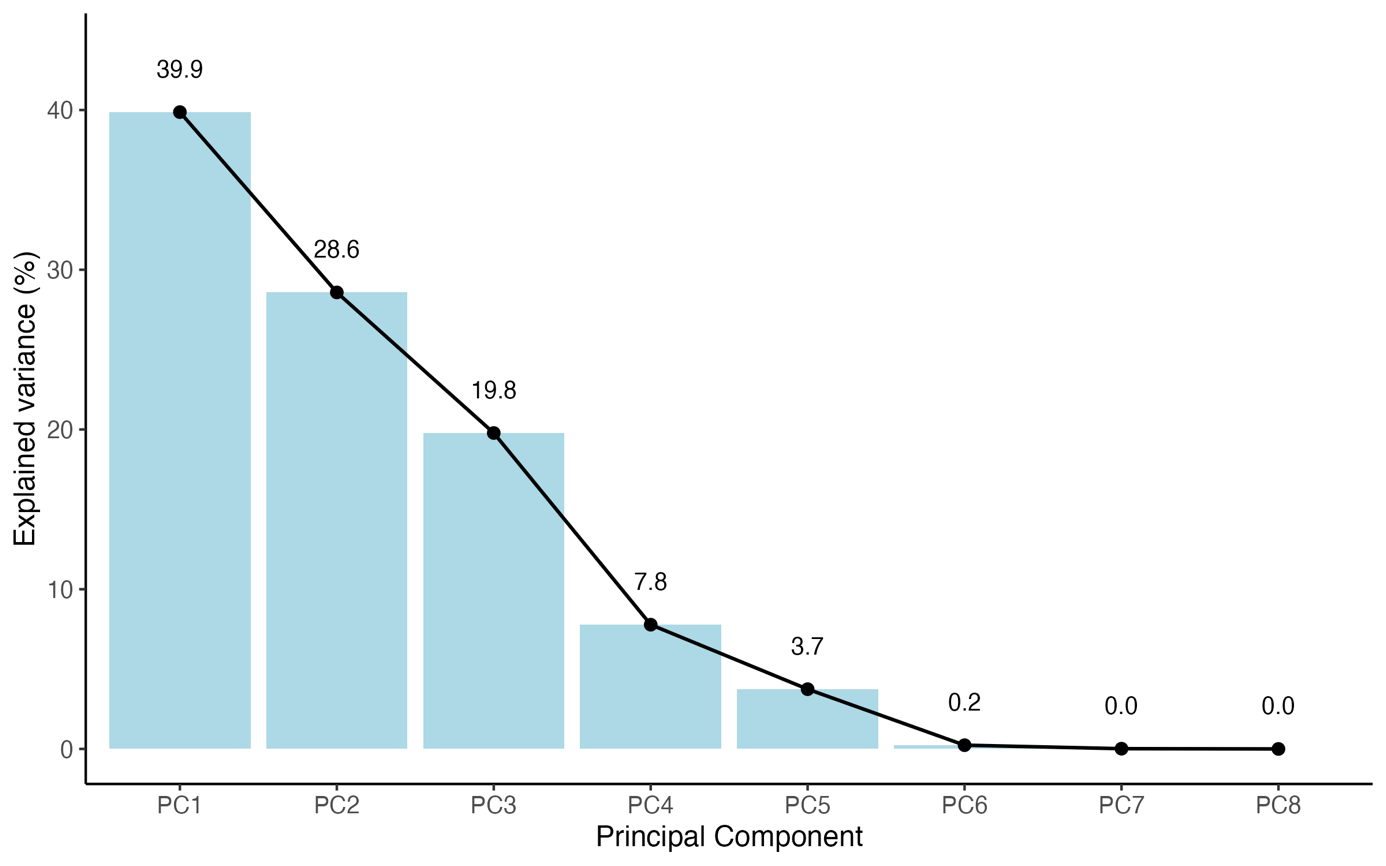
Figure 1.** Scree plot visualizing the variance explained by each principal component (PC) from the Principal Component Analysis (PCA).

| **Table 2.** Principal Component Analysis loadings for sleep parameters. | | | | | | | | |
| --- | --- | --- | --- | --- | --- | --- | --- | --- |
| **Variable** | **PC1** | **PC2** | **PC3** | **PC4** | **PC5** | **PC6** | **PC7** | **PC8** |
| Sleep onset | 0.11 | 0.17 | −0.74 | −0.23 | 0.09 | −0.03 | −0.60 | 0.00 |
| Sleep period time | −0.45 | −0.37 | 0.15 | −0.16 | −0.08 | 0.04 | −0.33 | −0.71 |
| Total sleep time | −0.25 | −0.57 | 0.14 | −0.15 | 0.11 | −0.28 | −0.29 | 0.63 |
| Wake after sleep onset | −0.48 | 0.30 | 0.07 | −0.06 | −0.38 | 0.63 | −0.16 | 0.33 |
| Number of awakenings | −0.40 | 0.34 | 0.11 | 0.02 | 0.84 | 0.04 | −0.01 | 0.00 |
| Sleep efficiency | 0.38 | −0.47 | −0.05 | 0.02 | 0.34 | 0.72 | −0.01 | 0.00 |
| Sleep offset | −0.35 | −0.21 | −0.52 | −0.37 | 0.00 | 0.02 | 0.65 | 0.00 |
| Sleep offset to breakfast time | 0.28 | 0.19 | 0.36 | −0.87 | 0.03 | 0.01 | 0.00 | 0.00 |

| **Table 3.** Variance inflation factor for predictors in the final linear mixed models. | |
| --- | --- |
| **Predictor** | **VIF** |
| Sleep offset | 1.55 |
| Sleep period time | 1.76 |
| Wake after sleep onset | 1.29 |
| Sex | 1.05 |
| Age | 1.04 |
| BMI | 1.04 |
| Abbreviations: BMI, body mass index; VIF, variance inflation factor. | |

| Table S1. STROBE checklist. | | | |
| --- | --- | --- | --- |
|  | Item | Recommendation | Page No. |
| **Title and abstract** | 1 | (*a*) Indicate the study’s design with a commonly used term in the title or the abstract | 3 |
|  |  | (*b*) Provide in the abstract an informative and balanced summary of what was done and what was found | 3 |
| Introduction | | | |
| Background/rationale | 2 | Explain the scientific background and rationale for the investigation being reported | 4–5 |
| Objectives | 3 | State specific objectives, including any prespecified hypotheses | 5 |
| Methods | | | |
| Study design | 4 | Present key elements of study design early in the paper | 6 |
| Setting | 5 | Describe the setting, locations, and relevant dates, including periods of recruitment, exposure, follow-up, and data collection | 6 |
| Participants | 6 | *Cross-sectional study*—Give the eligibility criteria, and the sources and methods of selection of participants | 6 |
| Variables | 7 | Clearly define all outcomes, exposures, predictors, potential confounders, and effect modifiers. Give diagnostic criteria, if applicable | 7–9 |
| Data sources/ measurement | 8 | For each variable of interest, give sources of data and details of methods of assessment (measurement). Describe comparability of assessment methods if there is more than one group | 6–8 |
| Bias | 9 | Describe any efforts to address potential sources of bias | 9–10 |
| Study size | 10 | Explain how the study size was arrived at | 9 |
| Quantitative variables | 11 | Explain how quantitative variables were handled in the analyses. If applicable, describe which groupings were chosen and why | 9–10 |
| Statistical methods | 12 | (*a*) Describe all statistical methods, including those used to control for confounding | 9–10 |
|  |  | (*b*) Describe any methods used to examine subgroups and interactions | 9–10 |
|  |  | (*c*) Explain how missing data were addressed | 9–10 |
|  |  | (*d*) *Cross-sectional study*—If applicable, describe analytical methods taking account of sampling strategy | NA |
|  |  | (*e*) Describe any sensitivity analyses | 10 |
| **Results** | | | |
| Participants | 13 | (a) Report numbers of individuals at each stage of study—e.g. numbers potentially eligible, examined for eligibility, confirmed eligible, included in the study, completing follow-up, and analysed | Figure S1 |
|  |  | (b) Give reasons for non-participation at each stage | Figure S1 |
|  |  | (c) Consider use of a flow diagram | Figure S1 |
| Descriptive data | 14 | (a) Give characteristics of study participants (eg demographic, clinical, social) and information on exposures and potential confounders | Table 1, Table S2 |
|  |  | (b) Indicate number of participants with missing data for each variable of interest | NA |
| Outcome data | 15 | *Cross-sectional study—*Report numbers of outcome events or summary measures | Table 1, Table S2 |
| Main results | 16 | (*a*) Give unadjusted estimates and, if applicable, confounder-adjusted estimates and their precision (eg, 95% confidence interval). Make clear which confounders were adjusted for and why they were included | 11–13 |
|  |  | (*b*) Report category boundaries when continuous variables were categorized | NA |
|  |  | (*c*) If relevant, consider translating estimates of relative risk into absolute risk for a meaningful time period | NA |
| Other analyses | 17 | Report other analyses done—e.g. analyses of subgroups and interactions, and sensitivity analyses | 10 |
| **Discussion** | | | |
| Key results | 18 | Summarise key results with reference to study objectives | 14 |
| Limitations | 19 | Discuss limitations of the study, taking into account sources of potential bias or imprecision. Discuss both direction and magnitude of any potential bias | 16 |
| Interpretation | 20 | Give a cautious overall interpretation of results considering objectives, limitations, multiplicity of analyses, results from similar studies, and other relevant evidence | 14–16 |
| Generalisability | 21 | Discuss the generalisability (external validity) of the study results | 16 |
| **Other information** | | | |
| Funding | 22 | Give the source of funding and the role of the funders for the present study and, if applicable, for the original study on which the present article is based | Title page |
| *Abbreviations*: NA, not applicable. | | | |

| **Table S2**. Main foods included in each food group category. | |
| --- | --- |
| **Food group** | **Main foods included** |
| Refined bread or pasta | White bread (including gluten-free): White bread, sliced bread, baguette, hot dog buns, hamburger buns.  Toasted bread, breadsticks (including gluten-free): Oatcakes, crispbreads (white and whole wheat), rice cakes, corn cakes, rusks.  Other breads (including gluten-free): Naan, garlic bread, tortillas, burritos, pita bread, rice wafers, olive oil bread. White pasta and rice, flours, grains: White pasta, white rice, couscous, gluten-free pasta. |
| Whole wheat bread or pasta | Whole wheat bread: Whole wheat bread, sliced, baguette, hot dog buns, hamburger buns.  Mixed, whole wheat, and seeded breads: Mixed grain bread, whole wheat or seeded bread, sliced, baguette, hot dog buns, hamburger buns.  Wholegrain pasta, brown rice, and other whole grains: Wholegrain pasta and rice, legume-based pasta, millet, quinoa. |
| Breakfast cereals with sugar | Oat-based cereal (sweetened)/granola or muesli: Breakfast cereals with oats or muesli (with or without nuts).  Other sweetened cereals: Sweetened breakfast cereals (with/without nuts), cornflakes, chocolate puffs, cereal bars. |
| Breakfast whole cereals | Bran or wholegrain cereals: Bran or wholegrain cereals, Weetabix, All-Bran flakes (with or without sugar), Special K.  Oatmeal/other unsweetened cereals: Porridge/oatmeal (including with added milk/nuts). |
| Pizza | Pizza (including gluten-free base). |
| Precooked dishes | Prepared pasta/rice dishes with added fat: Lasagna, instant noodles, croquettes, and burger meals.  Baby foods and purees.  Sushi. |
| Soups and purees | Homemade, powdered, or canned soups. |
| Whole dairy | Whole milk (>3.6 g fat/100 g): Cow, goat, sheep milk.  Whole yogurt: Yogurt made from whole milk, plain or flavored, petits suisses.  High-fat cheese: Cheese >17.5 g fat/100 g, including hard, soft, spreadable, blue cheese, feta, mozzarella, goat cheese, and others.  Cream: Dairy cream. |
| Skimmed and vegan dairy | Semi-skimmed milk (>1 g fat/100 g): Cow’s milk, other types.  Skimmed milk (<1 g fat/100 g): Cow’s milk, fortified/special milks (omega-3, folic acid, powdered milk).  Rice/oat and other plant-based drinks.  Soy drink: Soy-based beverages (including calcium-fortified).  Low-fat/skimmed yogurt: Fat-free and low-fat yogurt, plain or flavored.  Plant-based yogurts: Soy, oat, coconut, etc. yogurts.  Medium- and low-fat cheese: Cheese ≤17.5 g fat/100 g, including low-fat hard and spreadable cheese, cottage cheese. |
| Eggs | Whole eggs and processed egg dishes: Eggs, omelets, Scotch eggs, others. |
| Olive oil | Olive oil. |
| Butter and margarine | Butter and lard: Regular/light butter, dairy-based spreads.  Margarine.  Other oils and fats: Sunflower and seed oils, coconut oil. |
| Fresh or breaded chicken | Poultry: Poultry (with/without skin).  Breaded/battered/fried chicken: Fried/breaded chicken, stuffed chicken. |
| Pork, beef, lamb, or others | Pork.  Beef: Beef, venison.  Lamb: Lamb, goat, kid goat.  Other meats and offal: Other meats, including organ meats. |
| Processed meats | Sausages, burgers, bacon (lean or fatty), ham, liver pâté, meatballs (with/without sauce). |
| Fresh or coated seafood and white fish | White fish and canned tuna: Canned tuna, white fish, other fish.  Seafood: Prawns, lobster, crab, shellfish/crustaceans, mollusks, ceviche, surimi, fish roe.  Breaded/battered/processed fish: Fried/breaded fish, fish burgers, fish pâtés. |
| Blue fish | Oily fish, including salmon, sardines, mackerel, etc. (with/without sauce, canned). |
| Meat substitutes | Vegetarian meals: Quorn-based and vegetarian products.  Soy-based meals: Tofu products. |
| Fresh vegetables and legumes | Raw salad: Mixed salad, lettuce, watercress.  Cabbages, spinach: Broccoli, cabbage, kale, cauliflower, spinach, sprouts.  Root vegetables: Beetroot, carrots, celery, parsnip, turnip.  Tomatoes: Fresh and canned tomatoes.  *Allium* vegetables: Garlic, leek, onion.  Other vegetables (mushrooms, fruiting, mixed): Mushrooms, mixed vegetables, avocado, green beans, pumpkin, courgettes/zucchini, peppers, herbs, others.  Peas/sweetcorn: Peas, sweetcorn.  Legumes: Beans, lentils, chickpeas, broad beans, lupins.  Vegetable side dishes/preparations: Coleslaw, salads with added fat/mayonnaise, olives/pickles, seaweed, seafood salad.  Vegetable spreads/dips: Hummus, guacamole. |
| Boiled/mashed potatoes, or other tubers | Potatoes/sweet potatoes (baked/boiled): Potatoes, sweet potatoes, baked or boiled.  Mashed potatoes. |
| French fries | Fried/roasted potatoes: Processed potatoes (pre-fried or roasted) with added fat. |
| Fruits | Citrus fruits: Grapefruit, orange, mandarin, lemon.  Berries: Blackberries, strawberries, blueberries, raspberries, cherries.  Apples and pears.  Bananas and other fruits: Bananas, mixed fruits, grapes, mango, melon, peach, pineapple, kiwi, and others.  Dried fruit: Dried fruits, prunes. |
| Nuts | Salted nuts and seeds: Peanuts, pistachios, walnuts, etc., salted and/or roasted.  Unsalted nuts and seeds: Peanuts, pistachios, walnuts, etc., unsalted, raw and/or roasted. |
| Desserts, sweets, and jams | Added sugars and preserves: Table sugar, honey, preserves with sugar, cocoa powders (e.g., ColaCao), syrups. Chocolate sweets: Chocolate bars (milk, white, dark), chocolate-covered raisins, chocolate-covered sweets.  Other sweets: Hard and soft candies (including sugar-free), ice lollies/popsicles.  Biscuits/cookies: Chocolate biscuits, plain biscuits, sweet biscuits, cookies.  Milk/dairy desserts: Ice cream, milk puddings, dairy desserts, cheesecake, custard, flan.  Cakes, pastries, baked goods: Pancakes, croissants, pastries, filled buns, milk bread, fruit pies, cakes, donuts, other desserts, sweet snacks, sponge cake.  Soy-based desserts.  Nut-based spreads: Nutella, Nocilla, peanut butter (with added sugar). |
| Snacks | Potato crisps (chips), savory crackers, cheese snacks, and other savory biscuits. |
| Ketchup or mayonnaise sauces | High-fat sauces (oil-based): Mayonnaise, salad dressings, pesto, cheese sauce, white sauce, gravy, aioli.  Low-fat sauces: Ketchup, BBQ sauce, mustard, tomato sauce/purée, yeast extract, miso. |
| Juices | Orange, grapefruit juice, 100% fruit juice. |
| Water | Tap water, sparkling water. |
| Unsweetened beverages | Coffee, caffeinated: Instant, filter, cappuccino, espresso.  Coffee, decaffeinated: Instant decaf, filter, cappuccino, espresso.  Tea: Black, green, other teas.  Tea, decaffeinated: Decaf black tea, herbal teas, rooibos.  Low-/no-sugar soft drinks: Low-calorie fizzy drinks, sugar-free concentrated juice. |
| Sugared beverages | Sugary soft drinks carbonated and still: Regular fizzy drinks, concentrated juice, fruit smoothies.  Milk-/soy-based drinks and powders: Dairy-based milkshakes, milk drinks, hot chocolate, flavored shakes (chocolate, strawberry, etc.). |
| Alcoholic drinks* | White wine.  Red and rosé wine.  Beer and cider.  Spirits and other alcoholic beverages. |
| *Units in mL. | |

| **Table S3.** Dietary data of the study participants. | | | |
| --- | --- | --- | --- |
|  | **All** | **Men** | **Women** |
| *Sleep parameters from dinner-sleep observations (n = 178, 49% in women)** | | | |
| Energy (kcal) | 490.4 (333.8–709.3) | 550.5 (381.0–786.6) | 450.8 (310.6–640.7) |
| Fat (g) | 22.2 (12.4–34.5) | 23.8 (14.4–36.6) | 20.2 (10.6–30.5) |
| Saturated fat (g) | 6.7 (4.0–11.2) | 6.9 (4.4–12.0) | 6.6 (3.0–10.8) |
| Monounsaturated fat (g) | 8.0 (4.3–13.9) | 9.1 (5.0–14.4) | 6.3 (3.4–13.0) |
| Polyunsaturated fat (g) | 2.3 (1.2–4.2) | 2.5 (1.7–6.0) | 2.2 (0.8–3.6) |
| Cholesterol (mg) | 60.1 (21.3–122.9) | 60.4 (22.8–137.7) | 58.5 (19.8–112.4) |
| Protein (g) | 24.8 (13.8–36.1) | 26.7 (13.7–38.2) | 23.7 (14.0–34.4) |
| Carbohydrate (g) | 40.4 (24.0–64.3) | 44.1 (28.8–75.9) | 36.5 (22.5–60.2) |
| Sugars (g) | 8.6 (2.5–19.6) | 9.8 (2.6–21.0) | 6.8 (2.2–16.9) |
| Fiber (g) | 4.4 (2.5–6.4) | 4.2 (2.5–6.3) | 4.5 (2.5–6.5) |
| Alcohol (g) | 0.0 (0.0–0.0) | 0.0 (0.0–0.0) | 0.0 (0.0–0.0) |
| Fat (%) | 35.8 (17.9) | 37.3 (16.8) | 34.2 (18.9) |
| Monounsaturated fat (%) | 12.8 (8.6–23.7) | 14.0 (8.9–24.1) | 12.1 (8.0–22.6) |
| Polyunsaturated fat (%) | 4.3 (2.5–6.5) | 4.6 (2.8–6.7) | 4.1 (2.1–5.9) |
| Saturated fat (%) | 12.6 (8.1–18.5) | 11.9 (8.3–18.0) | 13.2 (7.2–18.5) |
| Protein (%) | 19.3 (13.6–23.4) | 18.1 (12.7–22.8) | 20.4 (15.9–26.1) |
| Carbohydrate (%) | 37.1 (27.1–48.3) | 36.5 (27.0–44.5) | 37.8 (27.1–51.6) |
| Sugars (%) | 6.3 (2.0–15.2) | 7.7 (2.0–15.0) | 5.9 (2.2–15.3) |
| Fiber density (g/1000 kcal) | 8.0 (5.2–12.5) | 7.1 (4.7–11.5) | 9.0 (6.2–17.1) |
| Alcohol (%) | 0.0 (0.0–0.0) | 0.0 (0.0–0.0) | 0.0 (0.0–0.0) |
| Carbohydrate to fiber ratio | 10.3 (6.8–13.6) | 10.6 (7.9–14.7) | 9.1 (4.8–13.1) |
| Carbohydrate to sugars ratio | 4.4 (1.7–9.6) | 3.7 (1.7–7.6) | 4.5 (1.7–11.6) |
| Refined bread or pasta (g) | 0.0 (0.0–60.0) | 0.0 (0.0–60.0) | 0.0 (0.0–60.0) |
| Whole wheat bread or pasta (g) | 0.0 (0.0–0.0) | 0.0 (0.0–0.0) | 0.0 (0.0–0.0) |
| Breakfast cereals with sugar (g) | 0.0 (0.0–0.0) | 0.0 (0.0–0.0) | 0.0 (0.0–0.0) |
| Breakfast whole cereals (g) | 0.0 (0.0) | 0.0 (0.0) | 0.0 (0.0) |
| Pizza (g) | 0.0 (0.0) | 0.0 (0.0) | 0.0 (0.0) |
| Precooked dishes (g) | 0.0 (0.0–0.0) | 0.0 (0.0–0.0) | 0.0 (0.0–0.0) |
| Soups and purees (g) | 0.0 (0.0–0.0) | 0.0 (0.0–0.0) | 0.0 (0.0–0.0) |
| Whole dairy (g) | 0.0 (0.0–25.0) | 0.0 (0.0–25.0) | 0.0 (0.0–21.3) |
| Skimmed and vegan dairy (g) | 0.0 (0.0–0.0) | 0.0 (0.0–0.0) | 0.0 (0.0–0.0) |
| Eggs (g) | 0.0 (0.0–0.0) | 0.0 (0.0–0.0) | 0.0 (0.0–0.0) |
| Olive oil (g) | 0.0 (0.0–0.0) | 0.0 (0.0–0.0) | 0.0 (0.0–0.0) |
| Butter and margarine (g) | 0.0 (0.0–0.0) | 0.0 (0.0–0.0) | 0.0 (0.0–0.0) |
| Fresh or breaded chicken (g) | 0.0 (0.0–0.0) | 0.0 (0.0–0.0) | 0.0 (0.0–0.0) |
| Pork, beef, lamb or others (g) | 0.0 (0.0–0.0) | 0.0 (0.0–0.0) | 0.0 (0.0–0.0) |
| Processed meats (g) | 0.0 (0.0–30.0) | 0.0 (0.0–30.3) | 0.0 (0.0–26.3) |
| Fresh or coated seafood and white fish (g) | 0.0 (0.0–0.0) | 0.0 (0.0–0.0) | 0.0 (0.0–0.0) |
| Blue fish (g) | 0.0 (0.0–0.0) | 0.0 (0.0–0.0) | 0.0 (0.0–0.0) |
| Meat substitutes (g) | 0.0 (0.0) | 0.0 (0.0) | 0.0 (0.0) |
| Fresh vegetables and legumes (g) | 0.0 (0.0–96.3) | 0.0 (0.0–100.0) | 0.0 (0.0–90.0) |
| Boiled potatoes, mashed potatoes, or other tubers (g) | 0.0 (0.0–0.0) | 0.0 (0.0–0.0) | 0.0 (0.0–0.0) |
| French fries (g) | 0.0 (0.0–0.0) | 0.0 (0.0–0.0) | 0.0 (0.0–0.0) |
| Fruits (g) | 0.0 (0.0–6.3) | 0.0 (0.0–0.0) | 0.0 (0.0–47.5) |
| Nuts (g) | 0.0 (0.0–0.0) | 0.0 (0.0–0.0) | 0.0 (0.0–0.0) |
| Desserts, sweets and jams (g) | 0.0 (0.0–0.0) | 0.0 (0.0–0.0) | 0.0 (0.0–0.0) |
| Snacks (g) | 0.0 (0.0–0.0) | 0.0 (0.0–0.0) | 0.0 (0.0–0.0) |
| Ketchup or mayonnaise sauces (g) | 0.0 (0.0–0.0) | 0.0 (0.0–0.0) | 0.0 (0.0–0.0) |
| Juices (g) | 0.0 (0.0) | 0.0 (0.0) | 0.0 (0.0) |
| Water (g) | 0.0 (0.0) | 0.0 (0.0) | 0.0 (0.0) |
| Unsweetened beverages (g) | 0.0 (0.0–0.0) | 0.0 (0.0–0.0) | 0.0 (0.0–0.0) |
| Sugared beverages (g) | 0.0 (0.0–0.0) | 0.0 (0.0–0.0) | 0.0 (0.0–0.0) |
| Alcoholic drinks (g) | 0.0 (0.0–0.0) | 0.0 (0.0–0.0) | 0.0 (0.0–0.0) |
| *Sleep parameters sleep-breakfast observations (n = 180, 48% in women)** | | | |
| Energy (kcal) | 354.9 (255.6–470.7) | 355.0 (236.4–467.4) | 354.9 (275.2–499.0) |
| Fat (g) | 15.1 (8.5–20.7) | 14.4 (7.4–20.3) | 15.8 (10.6–21.5) |
| Saturated fat (g) | 3.8 (1.9–7.3) | 3.6 (1.9–7.6) | 4.0 (2.0–7.0) |
| Monounsaturated fat (g) | 7.3 (3.1–10.2) | 6.2 (2.2–9.3) | 8.0 (3.8–11.2) |
| Polyunsaturated fat (g) | 1.4 (0.8–2.1) | 1.3 (0.6–1.9) | 1.6 (1.1–2.4) |
| Cholesterol (mg) | 16.9 (4.0–42.3) | 20.0 (4.0–47.0) | 14.0 (4.2–37.4) |
| Protein (g) | 12.3 (7.5–19.2) | 12.1 (6.8–18.6) | 13.4 (8.3–19.5) |
| Carbohydrate (g) | 41.4 (28.6–52.8) | 42.4 (24.1–53.5) | 40.5 (31.4–51.9) |
| Sugars (g) | 11.6 (5.2–17.9) | 12.2 (6.7–19.4) | 10.2 (4.6–17.3) |
| Fiber (g) | 3.2 (1.8–5.2) | 2.8 (1.8–4.2) | 3.6 (1.9–5.9) |
| Alcohol (g) | 0.0 (0.0–0.0) | 0.0 (0.0–0.0) | 0.0 (0.0–0.0) |
| Fat (%) | 36.7 (29.8–43.1) | 35.1 (29.1–41.3) | 37.6 (30.5–44.3) |
| Monounsaturated fat (%) | 15.7 (10.0–22.2) | 14.7 (9.1–21.1) | 16.5 (11.9–23.3) |
| Polyunsaturated fat (%) | 3.2 (2.7–4.5) | 2.9 (2.1–3.7) | 3.7 (2.9–5.0) |
| Saturated fat (%) | 10.0 (6.3–15.1) | 9.9 (5.8–16.8) | 10.0 (7.0–14.1) |
| Protein (%) | 15.3 (11.6–20.2) | 15.1 (10.8–19.7) | 15.5 (11.7–20.4) |
| Carbohydrate (%) | 46.1 (38.8–52.8) | 47.6 (38.9–53.5) | 45.2 (38.7–51.8) |
| Sugars (%) | 13.7 (8.2–23.3) | 14.7 (9.0–30.8) | 11.4 (6.4–19.0) |
| Fiber density (g/1000 kcal) | 8.4 (5.2–12.4) | 7.9 (5.2–11.0) | 9.3 (5.7–14.1) |
| Alcohol (%) | 0.0 (0.0–0.0) | 0.0 (0.0–0.0) | 0.0 (0.0–0.0) |
| Carbohydrate to fiber ratio | 13.5 (8.4–15.7) | 13.6 (9.9–16.1) | 11.6 (7.4–15.5) |
| Carbohydrate to sugars ratio | 3.3 (1.9–5.1) | 2.8 (1.5–4.6) | 3.6 (2.4–5.9) |
| Refined bread or pasta (g) | 0.0 (0.0–80.0) | 0.0 (0.0–80.0) | 0.0 (0.0–70.0) |
| Whole wheat bread or pasta (g) | 0.0 (0.0–0.0) | 0.0 (0.0–0.0) | 0.0 (0.0–52.5) |
| Breakfast cereals with sugar (g) | 0.0 (0.0–0.0) | 0.0 (0.0–0.0) | 0.0 (0.0–0.0) |
| Breakfast whole cereals (g) | 0.0 (0.0–0.0) | 0.0 (0.0–0.0) | 0.0 (0.0–0.0) |
| Pizza (g) | 0.0 (0.0) | 0.0 (0.0) | 0.0 (0.0) |
| Precooked dishes (g) | 0.0 (0.0) | 0.0 (0.0) | 0.0 (0.0) |
| Soups and purees (g) | 0.0 (0.0) | 0.0 (0.0) | 0.0 (0.0) |
| Whole dairy (g) | 0.0 (0.0–200.0) | 0.0 (0.0–200.0) | 0.0 (0.0–200.0) |
| Skimmed and vegan dairy (g) | 0.0 (0.0–37.5) | 0.0 (0.0–0.0) | 0.0 (0.0–100.0) |
| Eggs (g) | 0.0 (0.0–0.0) | 0.0 (0.0–0.0) | 0.0 (0.0–0.0) |
| Olive oil (g) | 0.0 (0.0–10.0) | 0.0 (0.0–10.0) | 5.0 (0.0–10.0) |
| Butter and margarine (g) | 0.0 (0.0–0.0) | 0.0 (0.0–0.0) | 0.0 (0.0–0.0) |
| Fresh or breaded chicken (g) | 0.0 (0.0–0.0) | 0.0 (0.0–0.0) | 0.0 (0.0–0.0) |
| Pork, beef, lamb or others (g) | 0.0 (0.0) | 0.0 (0.0) | 0.0 (0.0) |
| Processed meats (g) | 0.0 (0.0–0.0) | 0.0 (0.0–15.0) | 0.0 (0.0–0.0) |
| Fresh or coated seafood and white fish (g) | 0.0 (0.0–0.0) | 0.0 (0.0–0.0) | 0.0 (0.0–0.0) |
| Blue fish (g) | 0.0 (0.0–0.0) | 0.0 (0.0–0.0) | 0.0 (0.0–0.0) |
| Meat substitutes (g) | 0.0 (0.0) | 0.0 (0.0) | 0.0 (0.0) |
| Fresh vegetables and legumes (g) | 0.0 (0.0–30.0) | 0.0 (0.0–0.0) | 0.0 (0.0–40.0) |
| Boiled potatoes, mashed potatoes, or other tubers (g) | 0.0 (0.0) | 0.0 (0.0) | 0.0 (0.0) |
| French fries (g) | 0.0 (0.0) | 0.0 (0.0) | 0.0 (0.0) |
| Fruits (g) | 0.0 (0.0–0.0) | 0.0 (0.0–0.0) | 0.0 (0.0–0.0) |
| Nuts (g) | 0.0 (0.0–0.0) | 0.0 (0.0–0.0) | 0.0 (0.0–0.0) |
| Desserts, sweets and jams (g) | 0.0 (0.0–0.0) | 0.0 (0.0–0.8) | 0.0 (0.0–0.0) |
| Snacks (g) | 0.0 (0.0) | 0.0 (0.0) | 0.0 (0.0) |
| Ketchup or mayonnaise sauces (g) | 0.0 (0.0) | 0.0 (0.0) | 0.0 (0.0) |
| Juices (g) | 0.0 (0.0–0.0) | 0.0 (0.0–0.0) | 0.0 (0.0–0.0) |
| Water (g) | 0.0 (0.0) | 0.0 (0.0) | 0.0 (0.0) |
| Unsweetened beverages (g) | 100.0 (0.0–100.0) | 100.0 (0.0–100.0) | 100.0 (6.5–100.0) |
| Sugared beverages (g) | 0.0 (0.0) | 0.0 (0.0) | 0.0 (0.0) |
| Alcoholic drinks (g) | 0.0 (0.0) | 0.0 (0.0) | 0.0 (0.0) |
| Data are presented as mean (SD) when normally distributed or median (first quartile–third quartile) when not.  *Some participants contributed with two dinner-sleep and sleep-breakfast observations. | | | |


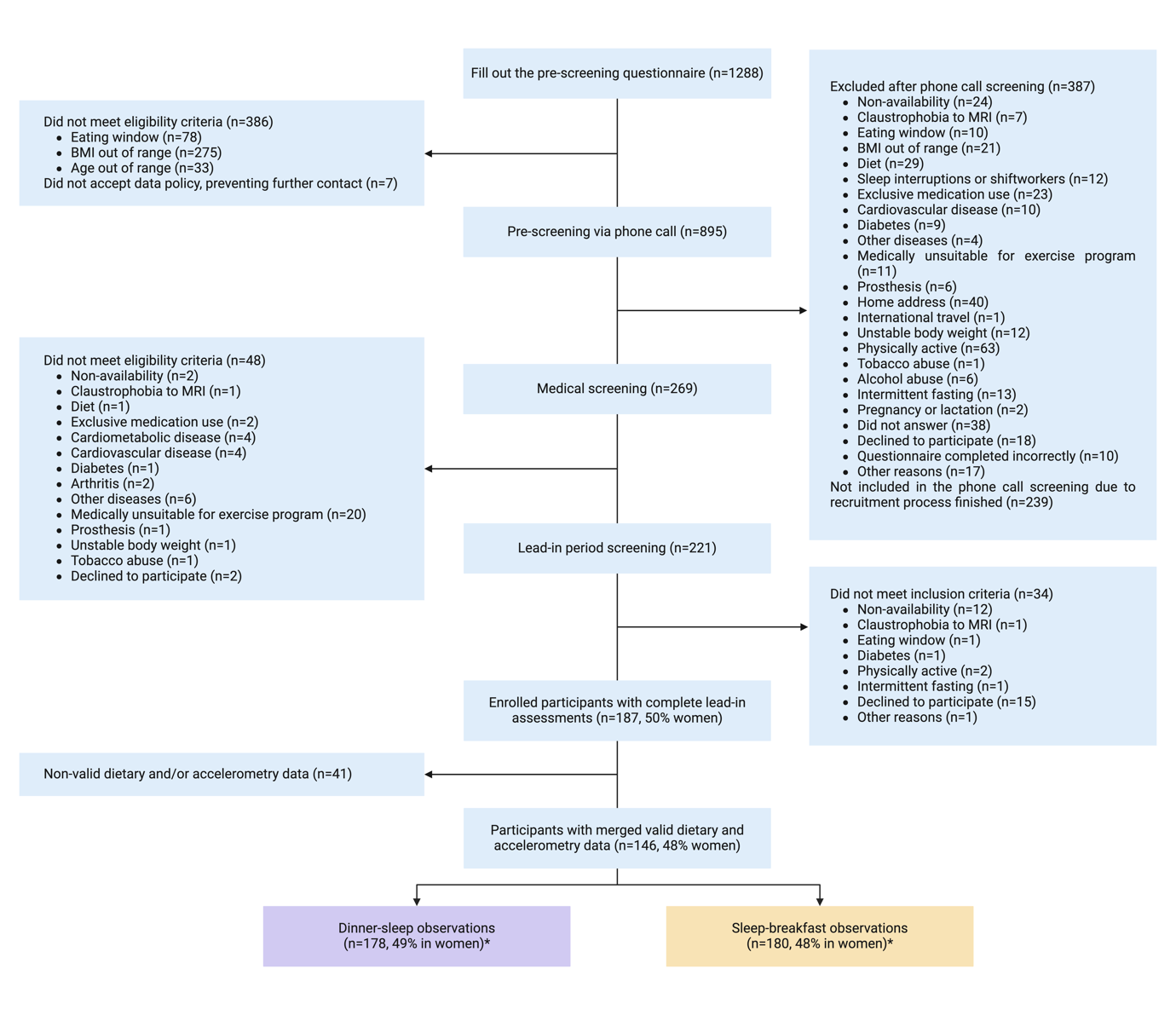


**Figure S1.** Study flow diagram. *Some participants contributed with two dinner-sleep and sleep-breakfast observations. *Abbreviations*: BMI, body mass index; MRI, magnetic resonance imaging.


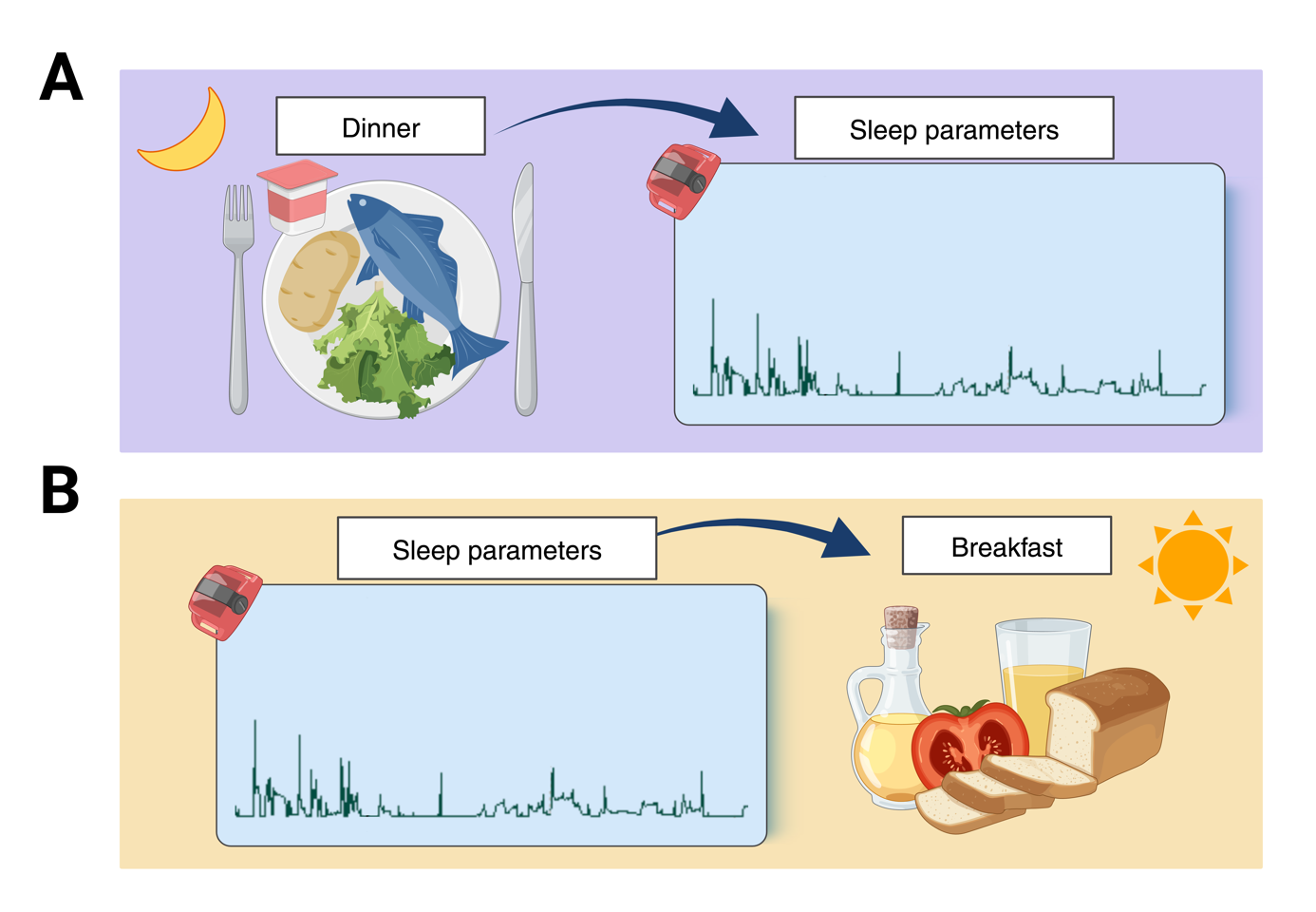


**Figure S2.** Overview of the study design. During the 2-week lead-in period, dinner dietary intake was paired with subsequent sleep parameters (Panel A), while sleep parameters were linked with subsequent breakfast dietary intake (Panel B) on a day-level basis in free-living adults with obesity. The waveforms depicted within the sleep parameters boxes illustrate representative accelerometer recordings captured throughout the night.

**
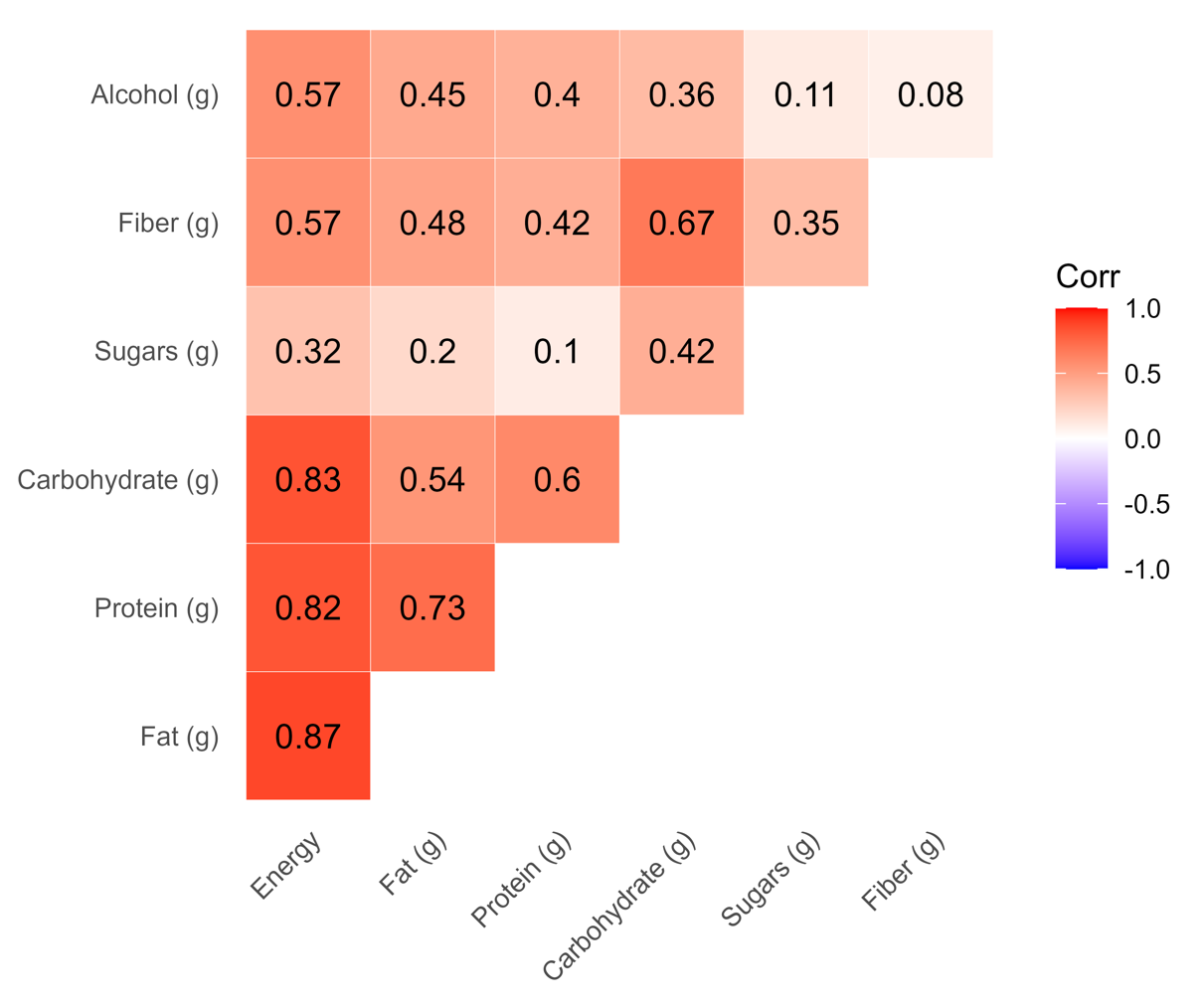
**

**Figure S3.** Bivariate correlations between energy and macronutrient intake at dinner. The colours of the squares represent the Spearman correlation coefficient. Red colours represent positive Spearman coefficients, whereas blue depicts negative coefficients. Numbers inside the squares represent Spearman correlation coefficients. *Abbreviations*: Corr, correlation coefficient.

**
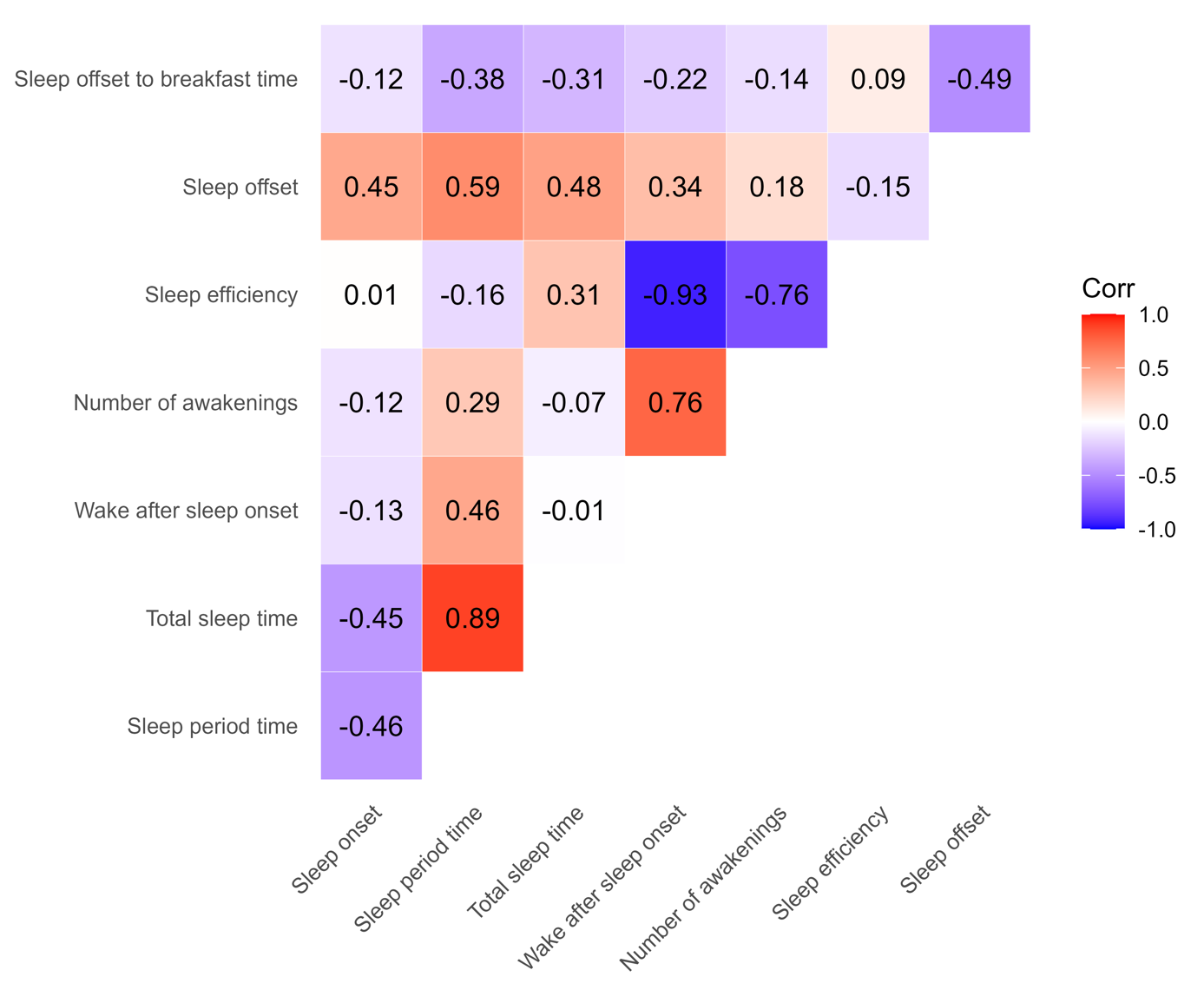
**

**Figure S4.** Bivariate correlations between sleep parameters before breakfast. The colours of the squares represent the Spearman correlation coefficient. Red colours represent positive Spearman coefficients, whereas blue depicts negative coefficients. Numbers inside the squares represent Spearman correlation coefficients. *Abbreviations*: Corr, correlation coefficient.

**
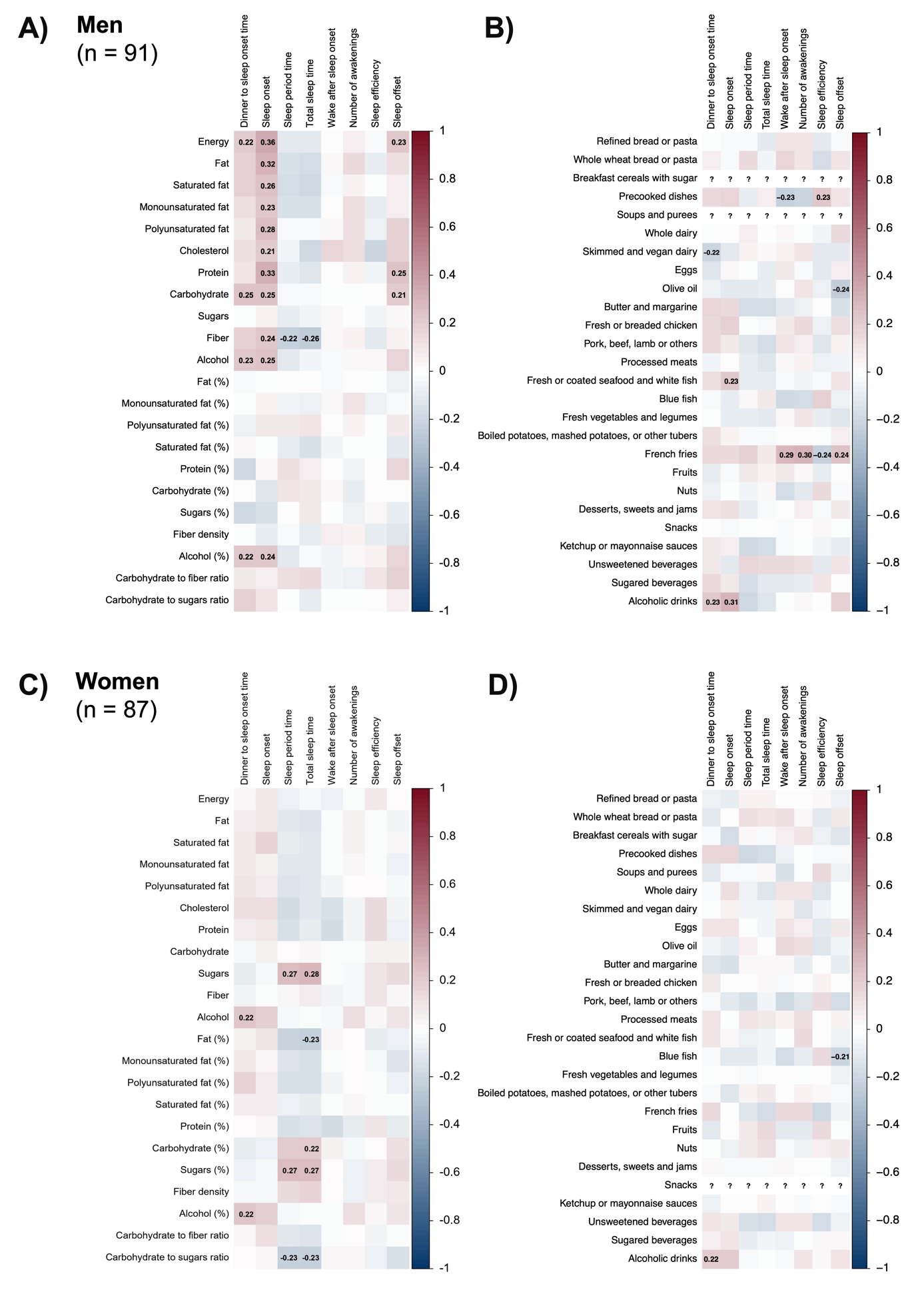
**

**Figure S5.** Bivariate correlations between nutrients and food groups intake at dinner with subsequent sleep parameters in men (panels A and B, respectively) and women (Panels C and D, respectively). The colours of the squares represent the Spearman correlation coefficient. Red colours represent positive Spearman coefficients, whereas blue depicts negative coefficients. Bold numbers inside the squares represent statistically significant Spearman correlation coefficients (*P* < 0.05). Question marks inside the squares represent the absence of data for associations between dinner and subsequent sleep parameters. *Abbreviations:* n, number of dinner-sleep observations.

**
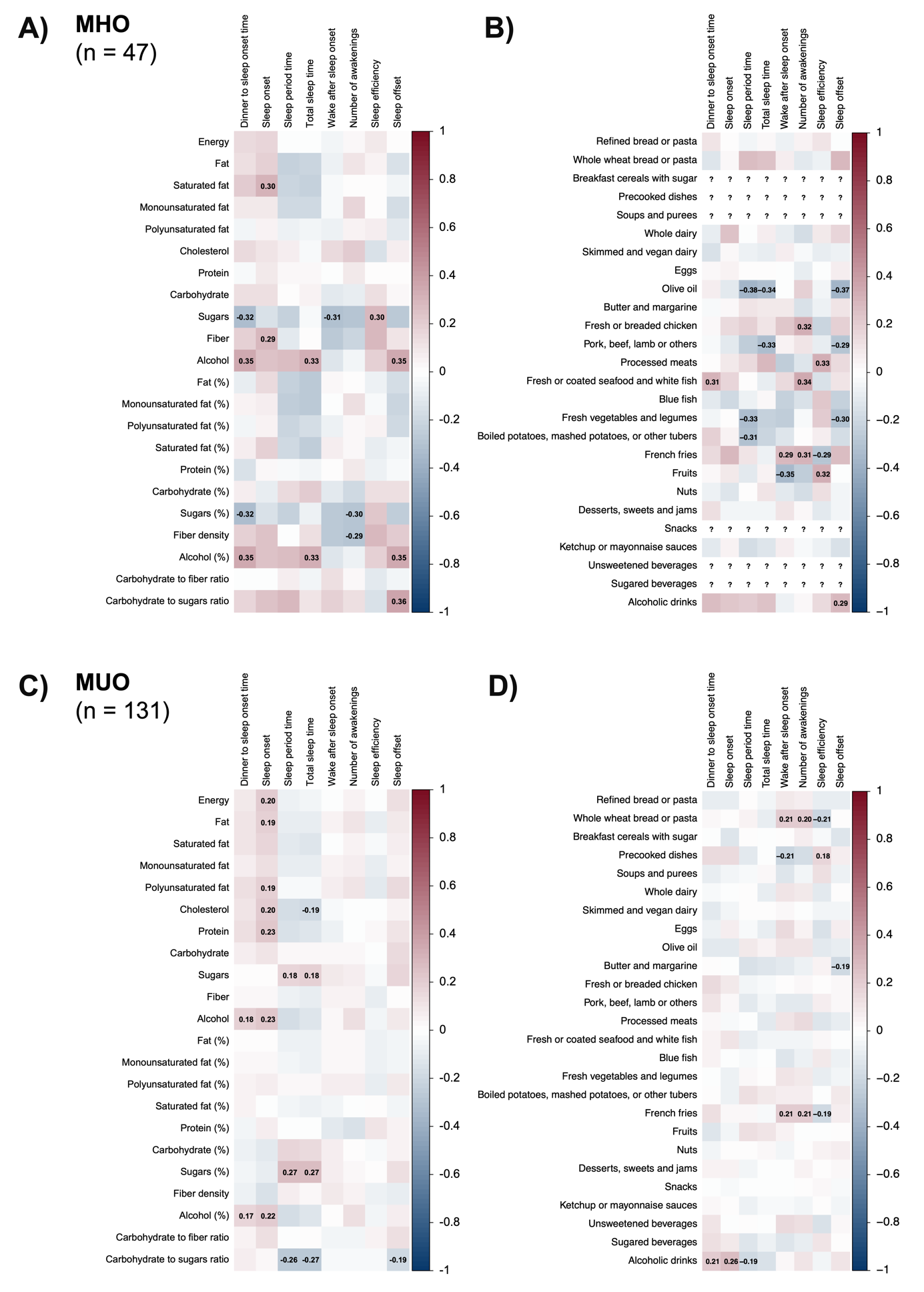
Figure S6.** Bivariate correlations between nutrients and food groups intake at dinner with subsequent sleep parameters in metabolically healthy participants with obesity (MHO; panels A and B, respectively) and metabolically unhealthy participants with obesity (MUO; panels C and D, respectively). The colours of the squares represent the Spearman correlation coefficient. Red colours represent positive Spearman coefficients, whereas blue depicts negative coefficients. Bold numbers inside the squares represent statistically significant Spearman correlation coefficients (*P* < 0.05). Question marks inside the squares represent the absence of data for associations between dinner and subsequent sleep parameters. *Abbreviations:* n, number of dinner-sleep observations.

**
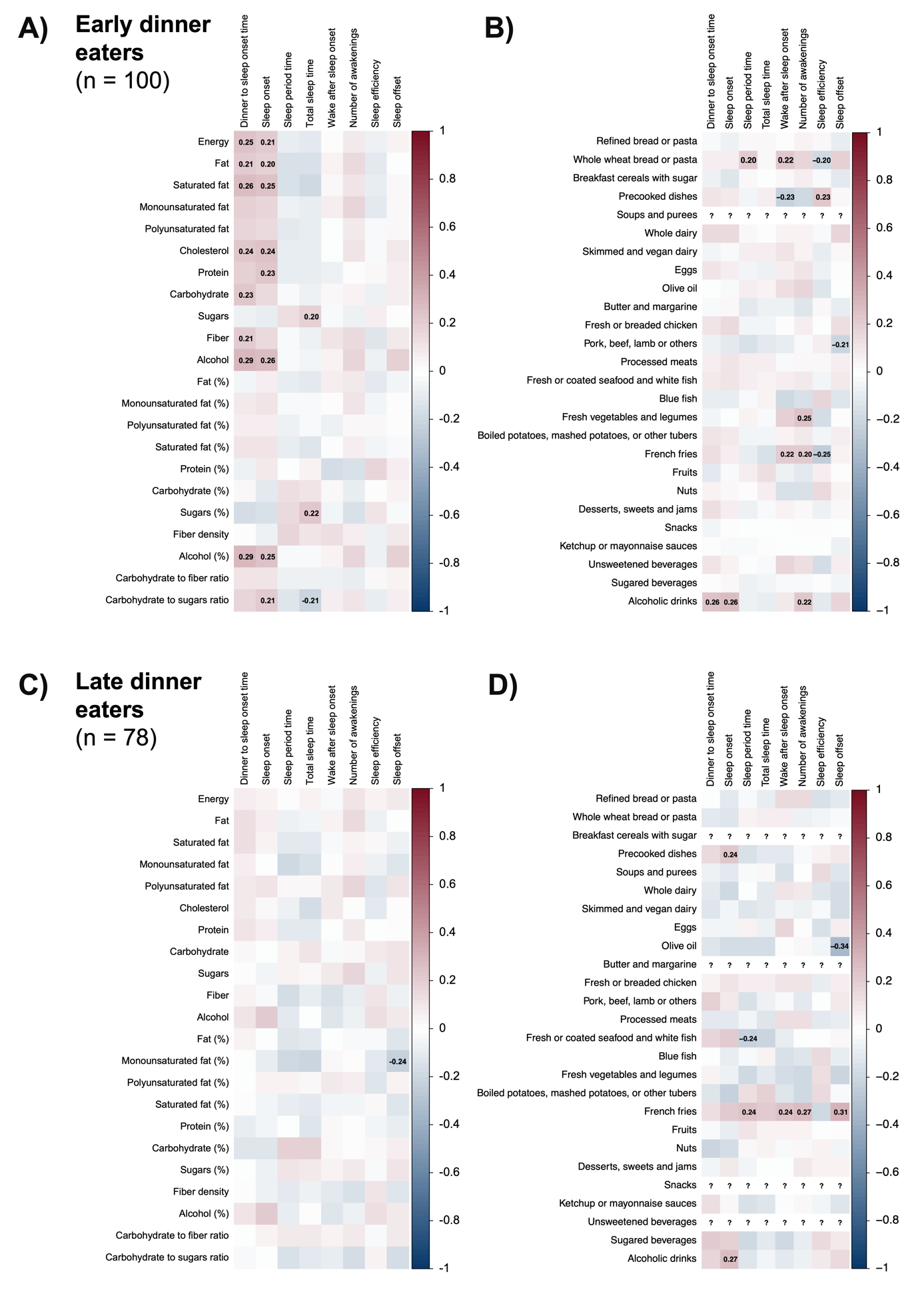
**

**Figure S7.** Bivariate correlations between nutrients and food groups intake at dinner with subsequent sleep parameters in early dinner eaters (dinner earlier than 21:30; panels A and B, respectively) and late dinner eaters (dinner later than 21:30; panels C and D, respectively). The colours of the squares represent the Spearman correlation coefficient. Red colours represent positive Spearman coefficients, whereas blue depicts negative coefficients. Bold numbers inside the squares represent statistically significant Spearman correlation coefficients (*P* < 0.05). Question marks inside the squares represent the absence of data for associations between dinner and subsequent sleep parameters. *Abbreviations:* n, number of dinner-sleep observations.

**
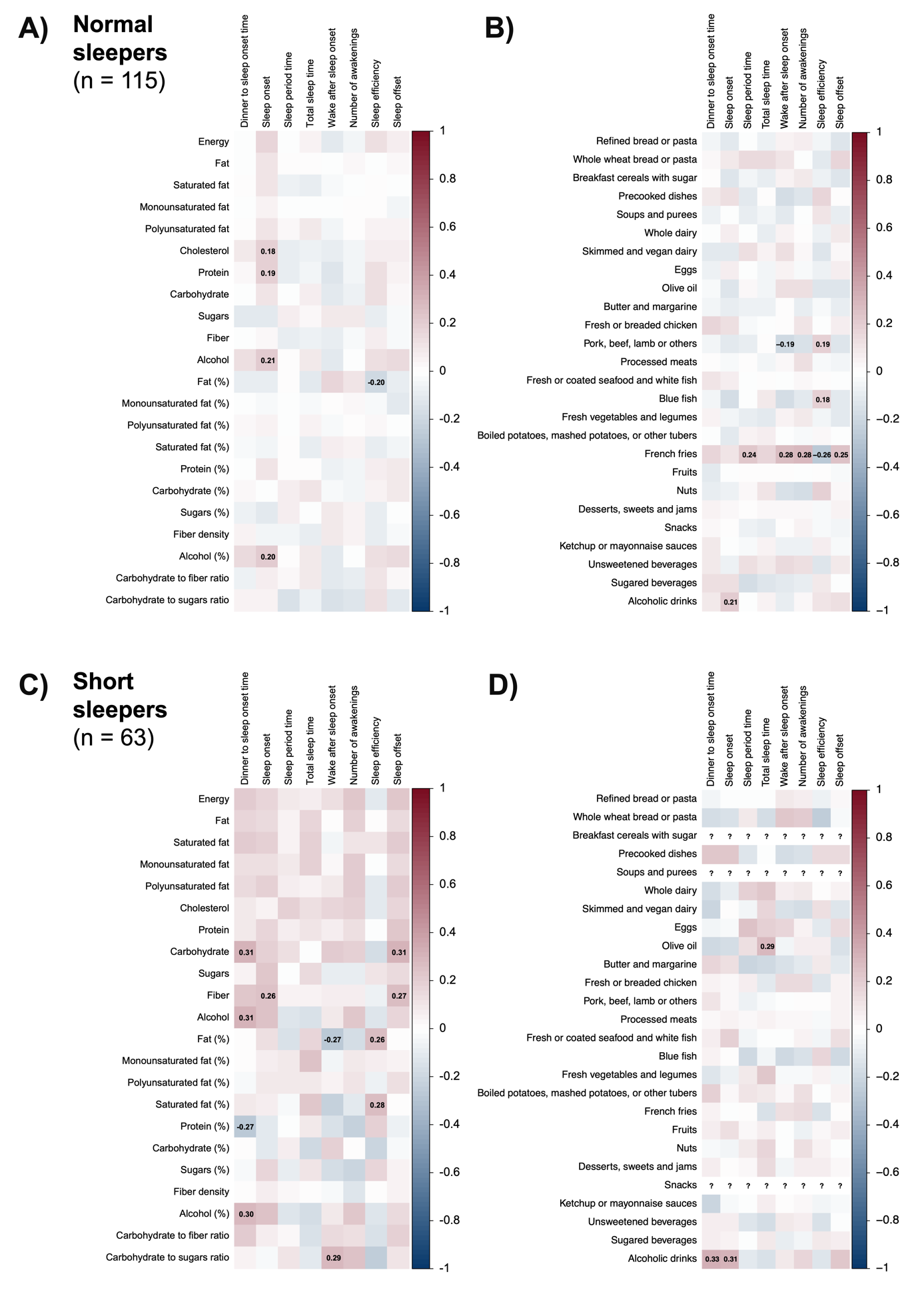
**

**Figure S8.** Bivariate correlations between nutrients and food groups intake at dinner with subsequent sleep parameters in normal sleepers (total sleep time ≥ 6 h; panels A and B, respectively) and short sleepers (total sleep time < 6 h; panels C and D, respectively). The colours of the squares represent the Spearman correlation coefficient. Red colours represent positive Spearman coefficients, whereas blue depicts negative coefficients. Bold numbers inside the squares represent statistically significant Spearman correlation coefficients (*P* < 0.05). Question marks inside the squares represent the absence of data for associations between dinner and subsequent sleep parameters. *Abbreviations:* n, number of dinner-sleep observations.

**
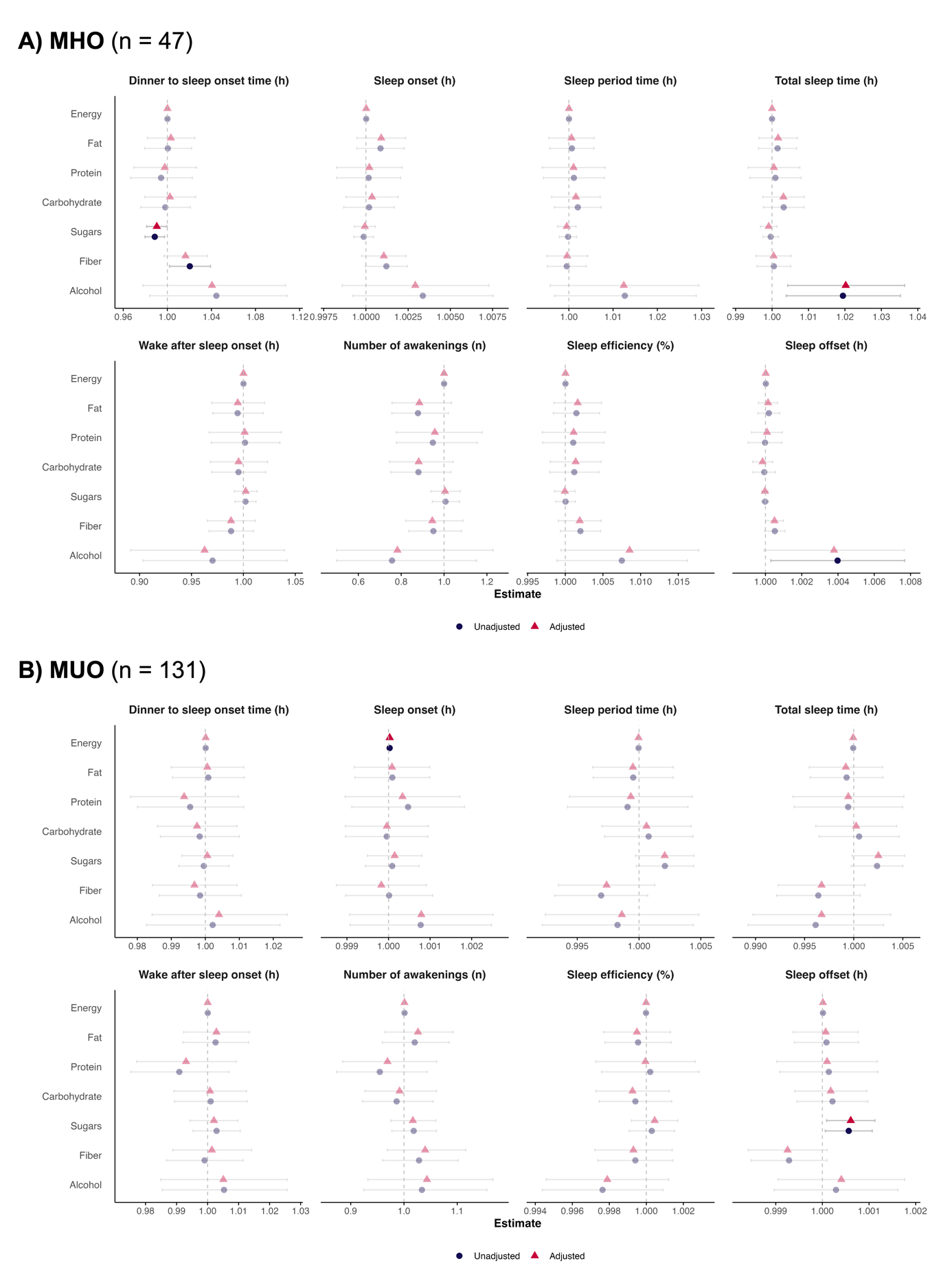
**

**Figure S9.** Forest plots of associations of dinner energy intake and macronutrient intake with subsequent sleep parameters in metabolically healthy participants with obesity (MHO, panel A), and metabolically unhealthy participants with obesity (MUO, panel B). Dietary data were imputed using the nutrient density model, as described elsewhere [3]. Estimates and 95% confidence intervals (CIs) were obtained via linear mixed models. Blue circles and their corresponding 95% CIs represent estimates from unadjusted models, whereas red triangles and their corresponding 95% CIs represent estimates from models adjusted for age, sex, body mass index, and moderate-to-vigorous physical activity. Sleep parameters were log10-transformed for statistical analyses; however, results were back-transformed to improve interpretability. Vivid symbols and 95% CIs indicate significant estimates (*P* < 0.05). *Abbreviations:* n, number of dinner-sleep observations.

**
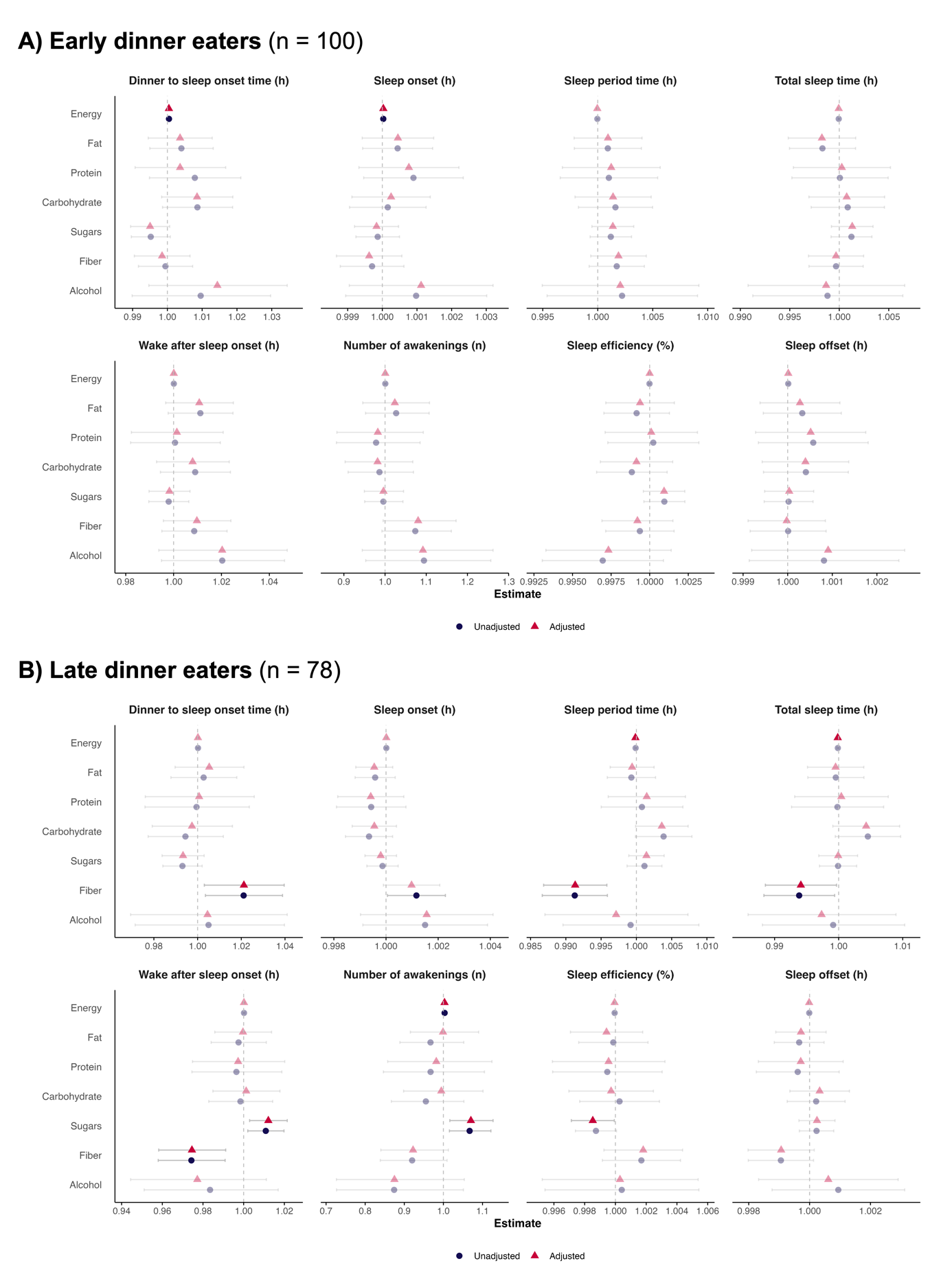
**

**Figure S10.** Forest plots of associations of dinner energy intake and macronutrient intake with subsequent sleep parameters in early dinner eaters (dinner earlier than 21:30; panel A), and late dinner eaters (dinner later than 21:30; panel B). Dietary data were imputed using the nutrient density model, as described elsewhere [3]. Estimates and 95% confidence intervals (CIs) were obtained via linear mixed models. Blue circles and their corresponding 95% CIs represent estimates from unadjusted models, whereas red triangles and their corresponding 95% CIs represent estimates from models adjusted for age, sex, body mass index, and moderate-to-vigorous physical activity. Sleep parameters were log10-transformed for statistical analyses; however, results were back-transformed to improve interpretability. Vivid symbols and 95% CIs indicate significant estimates (*P* < 0.05). *Abbreviations:* n, number of dinner-sleep observations.

**
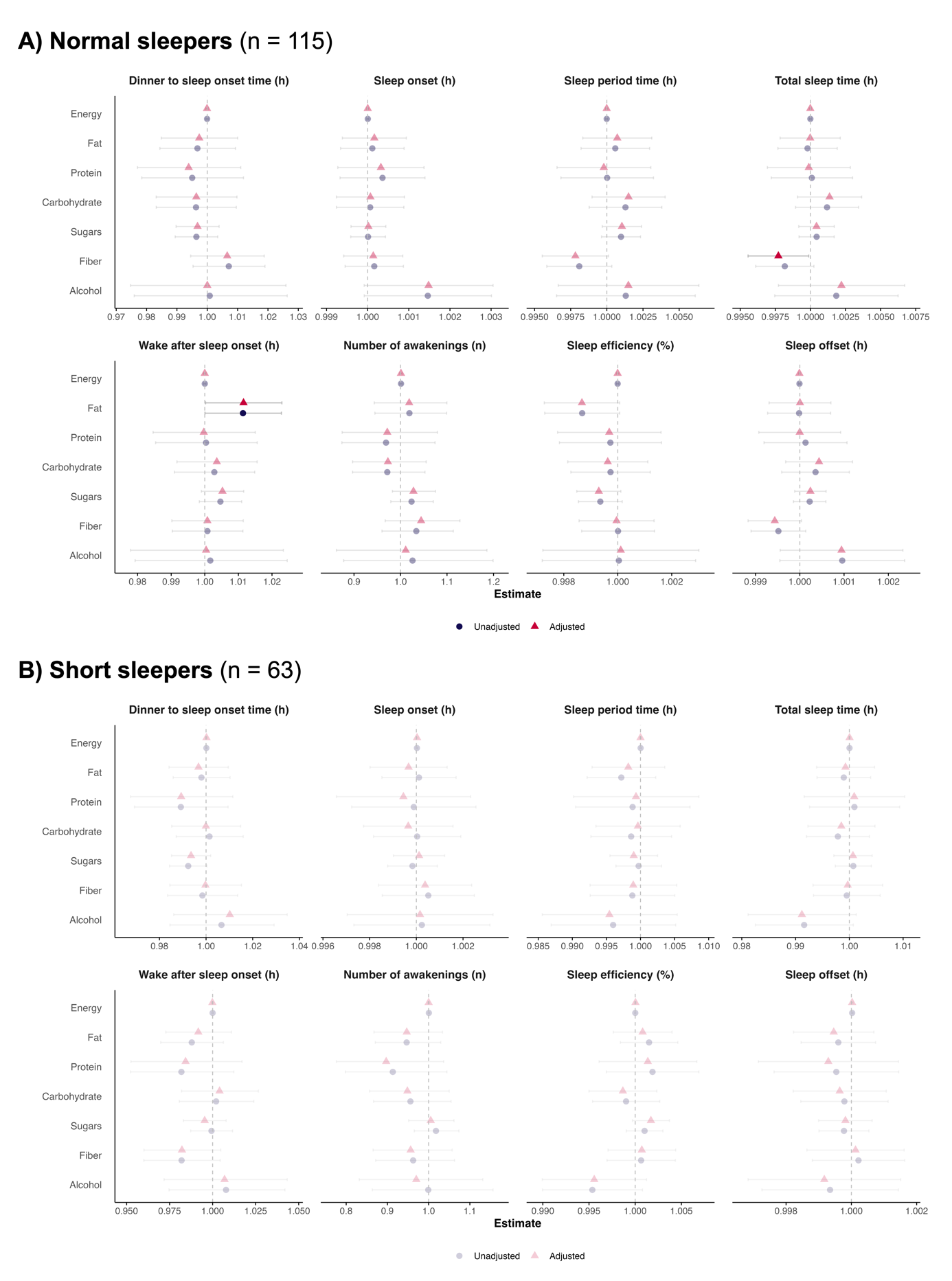
**

**Figure S11.** Forest plots of associations of dinner energy intake and macronutrient intake with subsequent sleep parameters in normal sleepers (total sleep time ≥ 6 h; panel A), and short sleepers (total sleep time < 6 h; panel B). Dietary data were imputed using the nutrient density model, as described elsewhere [3]. Estimates and 95% confidence intervals (CIs) were obtained via linear mixed models. Blue circles and their corresponding 95% CIs represent estimates from unadjusted models, whereas red triangles and their corresponding 95% CIs represent estimates from models adjusted for age, sex, body mass index, and moderate-to-vigorous physical activity. Sleep parameters were log10-transformed for statistical analyses; however, results were back-transformed to improve interpretability. Vivid symbols and 95% CIs indicate significant estimates (*P* < 0.05). *Abbreviations:* n, number of dinner-sleep observations.

**
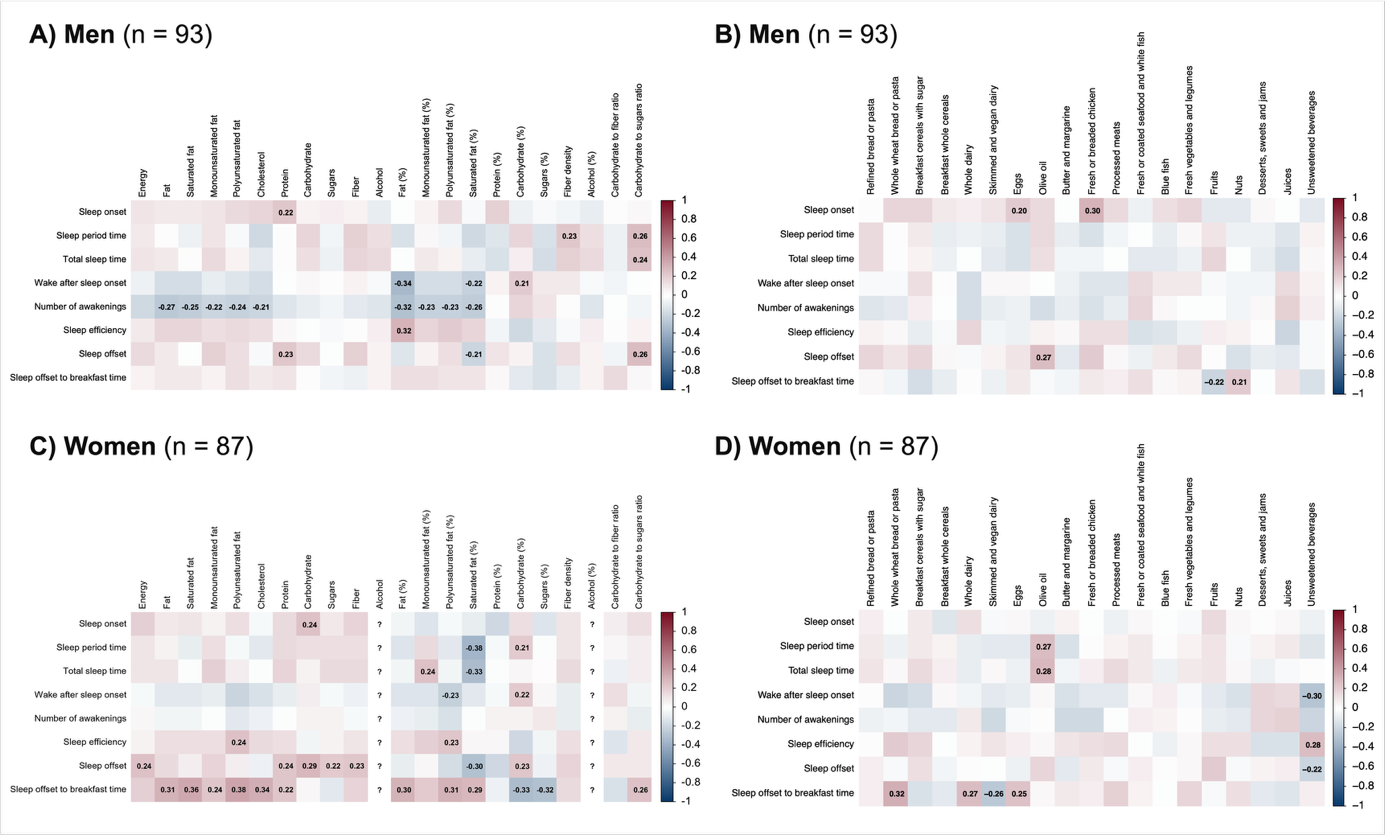
**

**Figure S12.** Bivariate correlations between sleep parameters and nutrients and food groups intake at subsequent breakfast in men (panels A and B, respectively) and women (Panels C and D, respectively). The colours of the squares represent the Spearman correlation coefficient. Red colours represent positive Spearman coefficients, whereas blue depicts negative coefficients. Bold numbers inside the squares represent statistically significant Spearman correlation coefficients (*P* < 0.05). Question marks inside the squares represent the absence of data for associations between dinner and subsequent sleep parameters. *Abbreviations:* n, number of sleep-breakfast observations.

**
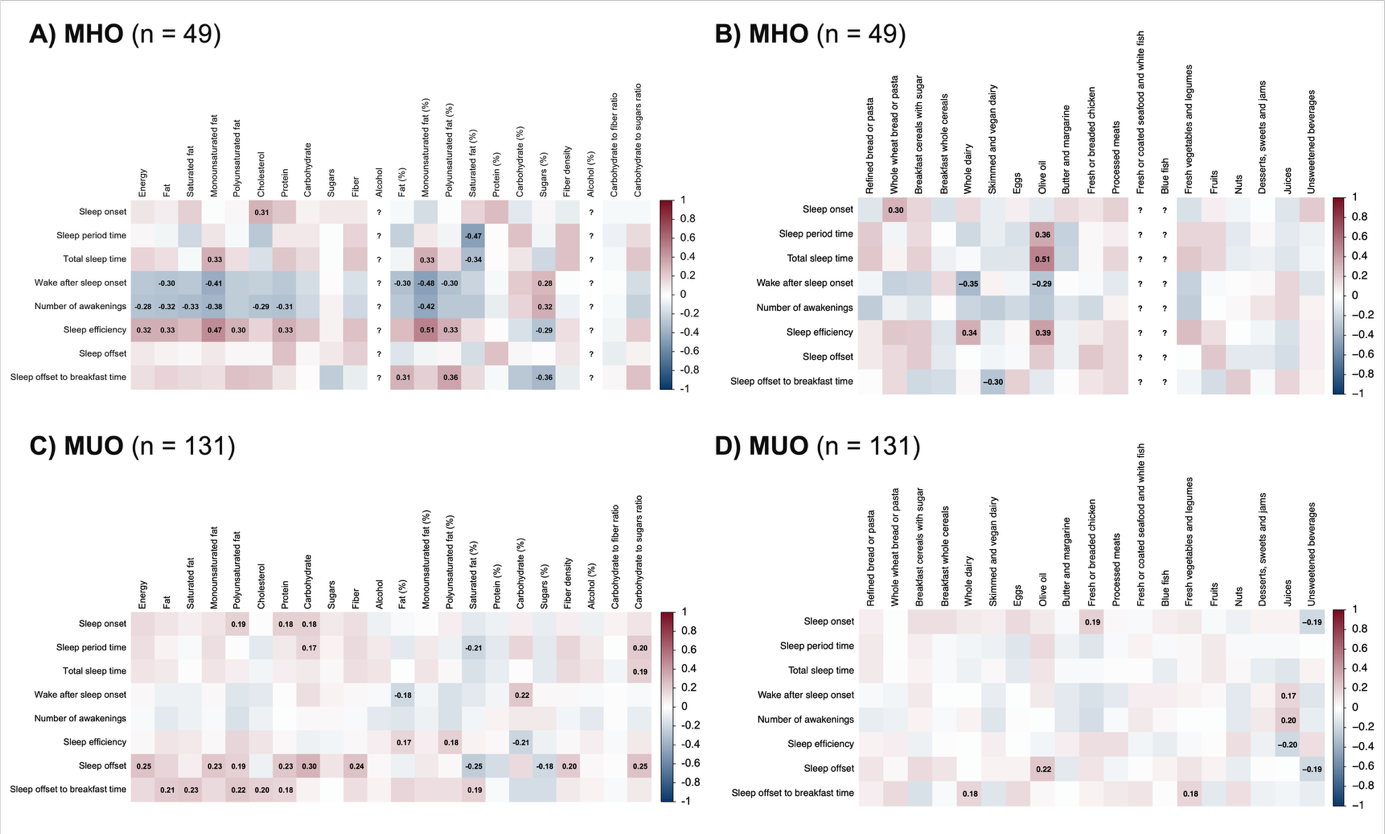
**

**Figure S13.** Bivariate correlations between sleep parameters and nutrients and food groups intake at subsequent breakfast in metabolically healthy participants with obesity (MHO; panels A and B, respectively) and metabolically unhealthy participants with obesity (MUO; panels C and D, respectively). The colours of the squares represent the Spearman correlation coefficient. Red colours represent positive Spearman coefficients, whereas blue depicts negative coefficients. Bold numbers inside the squares represent statistically significant Spearman correlation coefficients (*P* < 0.05). Question marks inside the squares represent the absence of data for associations between dinner and subsequent sleep parameters. *Abbreviations:* n, number of sleep-breakfast observations.

**
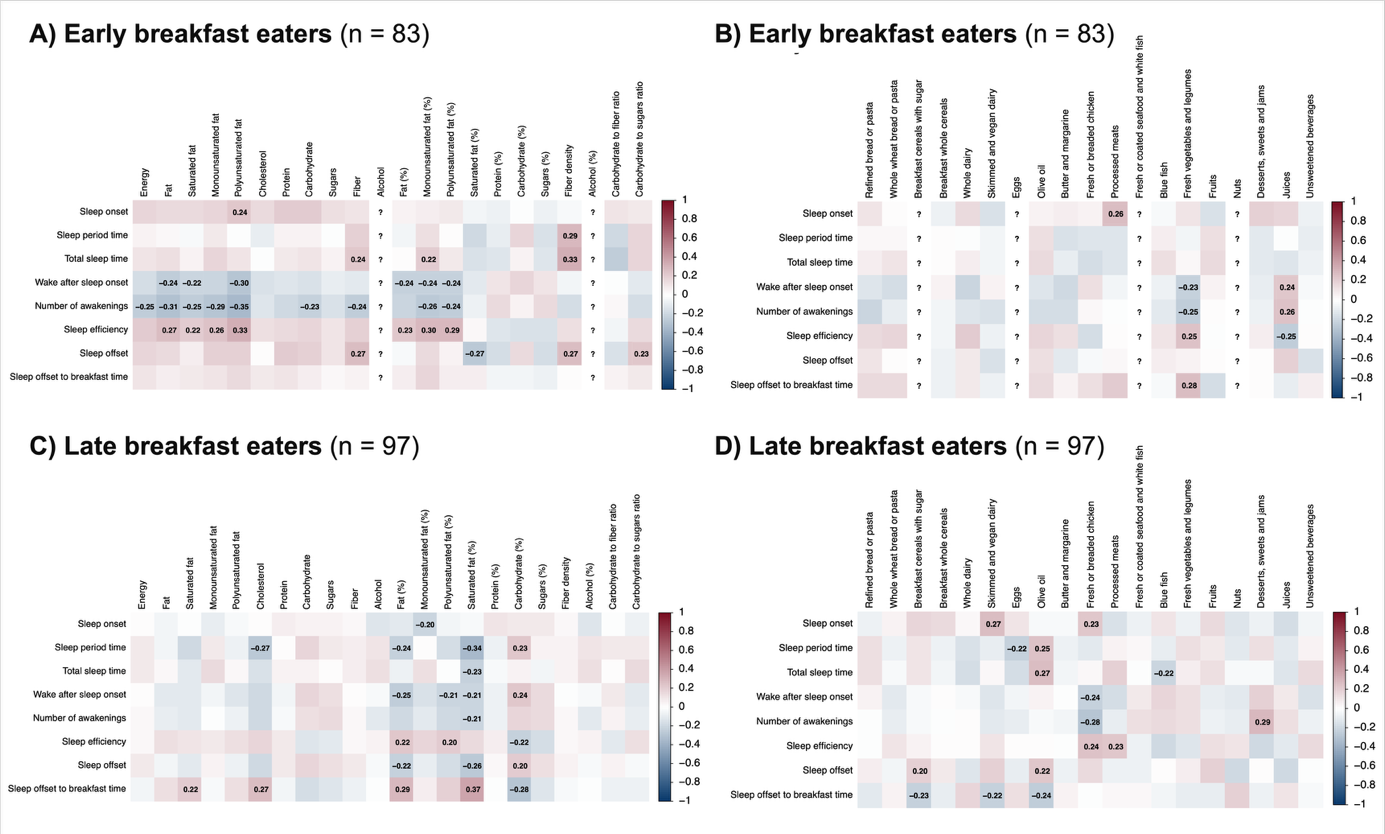
**

**Figure S14.** Bivariate correlations between sleep parameters and nutrients and food groups intake at subsequent breakfast in early breakfast eaters (breakfast earlier than 9:00; panels A and B, respectively) and late breakfast eaters (breakfast later than 9:00; panels C and D, respectively). The colours of the squares represent the Spearman correlation coefficient. Red colours represent positive Spearman coefficients, whereas blue depicts negative coefficients. Bold numbers inside the squares represent statistically significant Spearman correlation coefficients (*P* < 0.05). Question marks inside the squares represent the absence of data for associations between dinner and subsequent sleep parameters. *Abbreviations:* n, number of sleep-breakfast observations.

**
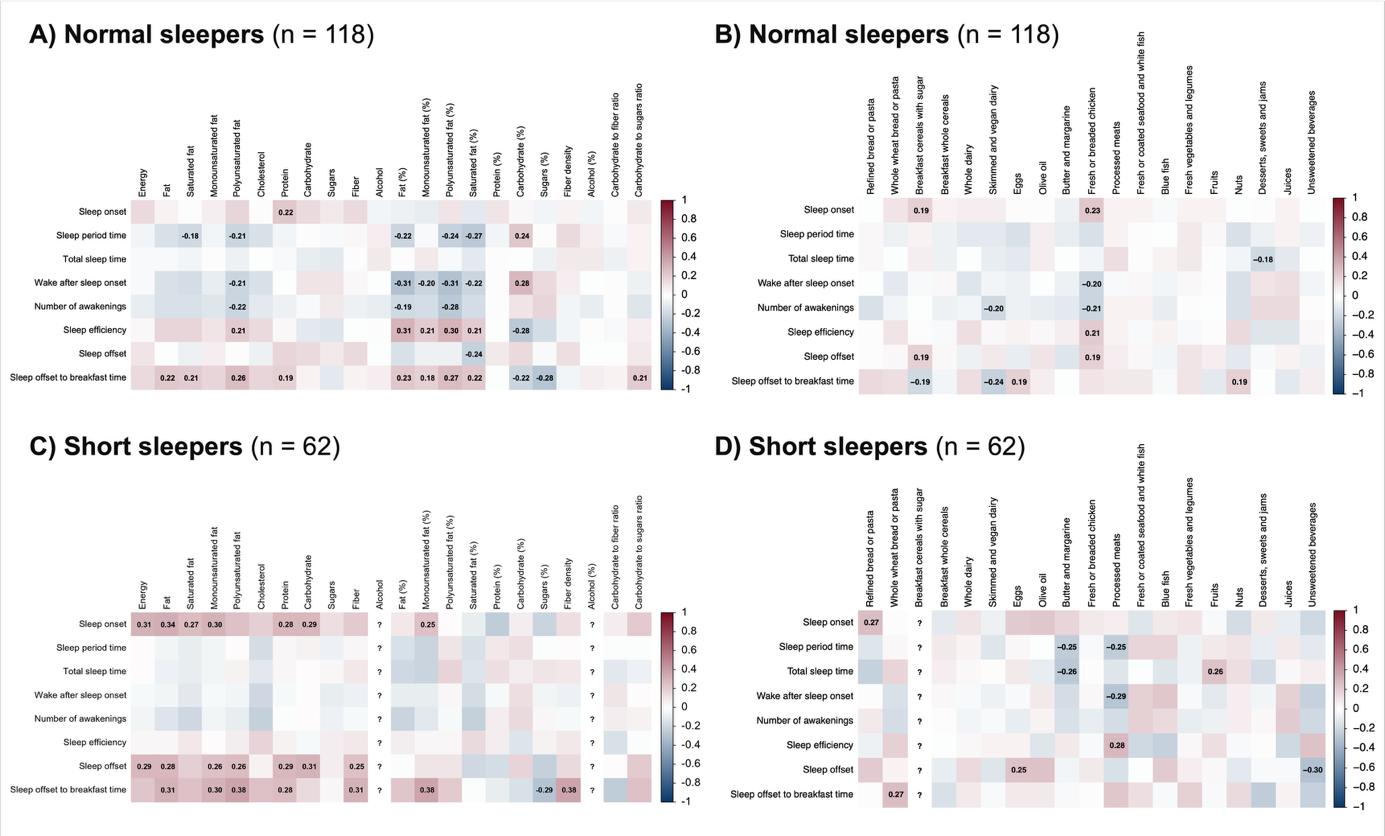
**

**Figure S15.** Bivariate correlations between sleep parameters and nutrients and food groups intake at subsequent breakfast in normal sleepers (total sleep time ≥ 6 h; panels A and B, respectively) and short sleepers (total sleep time < 6 h; panels C and D, respectively). The colours of the squares represent the Spearman correlation coefficient. Red colours represent positive Spearman coefficients, whereas blue depicts negative coefficients. Bold numbers inside the squares represent statistically significant Spearman correlation coefficients (*P* < 0.05). Question marks inside the squares represent the absence of data for associations between dinner and subsequent sleep parameters. *Abbreviations:* n, number of sleep-breakfast observations.

**
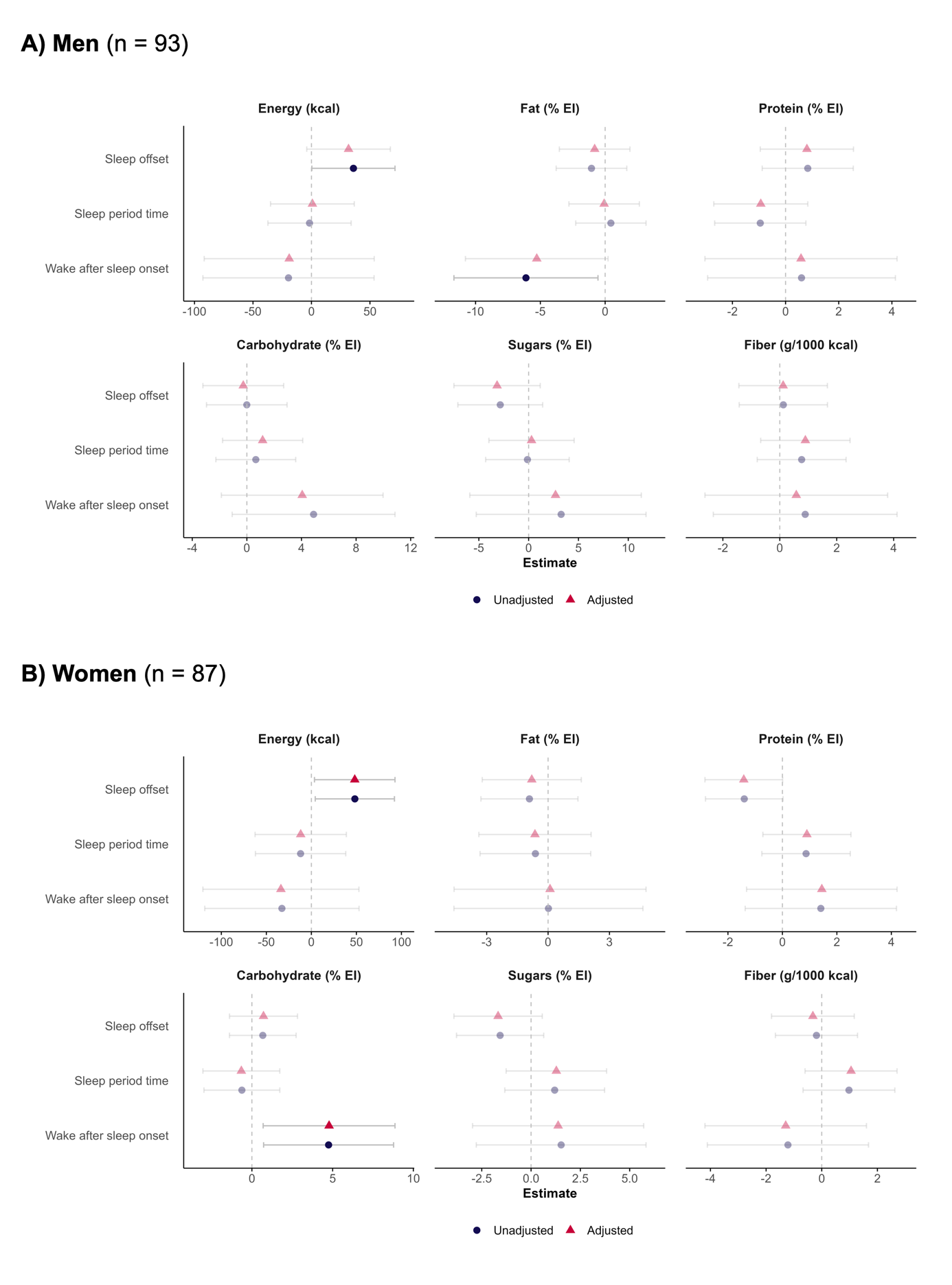
**

**Figure S16.** Forest plots of associations of sleep parameters with subsequent breakfast energy intake and macronutrient intake in men (panel A) and women (panel B). Dietary data were imputed using the nutrient density model, as described elsewhere [3]. Estimates and 95% confidence intervals (CIs) were obtained via linear mixed models. Blue circles and their corresponding 95% CIs represent estimates from unadjusted models, whereas red triangles and their corresponding 95% CIs represent estimates from models adjusted for age, sex, and body mass index. Vivid symbols and 95% CIs indicate significant estimates (*P* < 0.05). *Abbreviations:* n, number of sleep-breakfast observations.

**References**

1 Friedewald WT, Levy RI, Fredrickson DS. Estimation of the concentration of low-density lipoprotein cholesterol in plasma, without use of the preparative ultracentrifuge. *Clin Chem*. 1972;18:499–502.

2 Stergiou GS, Palatini P, Parati G, *et al.* 2021 European Society of Hypertension practice guidelines for office and out-of-office blood pressure measurement. *J Hypertens*. 2021;39:1293–302. doi: 10.1097/HJH.0000000000002843

3 Tomova GD, Arnold KF, Gilthorpe MS, *et al.* Adjustment for energy intake in nutritional research: a causal inference perspective. *American Journal of Clinical Nutrition*. 2022;115:189–98. doi: 10.1093/ajcn/nqab266
